# Comparative phylogenomics of the CBL-CIPK calcium-decoding network in the moss *Physcomitrella*, Arabidopsis, and other green lineages

**DOI:** 10.3389/fpls.2014.00187

**Published:** 2014-05-14

**Authors:** Thomas J. Kleist, Andrew L. Spencley, Sheng Luan

**Affiliations:** ^1^Department of Plant and Microbial Biology, University of California BerkeleyBerkeley, CA, USA; ^2^Department of Dermatology, Stanford UniversityStanford, CA, USA

**Keywords:** CBL-CIPK, calcium signaling, plant abiotic stress physiology, plant nutrition, evolution, molecular

## Abstract

Land plants have evolved a host of anatomical and molecular adaptations for terrestrial growth. Many of these adaptations are believed to be elaborations of features that were present in their algal-like progenitors. In the model plant Arabidopsis, 10 Calcineurin B-Like proteins (CBLs) function as calcium sensors and modulate the activity of 26 CBL-Interacting Protein Kinases (CIPKs). The CBL-CIPK network coordinates environmental responses and helps maintain proper ion balances, especially during abiotic stress. We identified and analyzed CBL and CIPK homologs in green lineages, including CBLs and CIPKs from charophyte green algae, the closest living relatives of land plants. Phylogenomic evidence suggests that the network expanded from a small module, likely a single CBL-CIPK pair, present in the ancestor of modern plants and algae. Extreme conservation of the NAF motif, which mediates CBL-CIPK physical interactions, among all identified CIPKs supports the interpretation of CBL and CIPK homologs in green algae and early diverging land plants as functionally linked network components. We identified the full complement of CBL and CIPK loci in the genome of *Physcomitrella*, a model moss. These analyses demonstrate the strong effects of a recent moss whole genome duplication: CBL and CIPK loci appear in cognate pairs, some of which appear to be pseudogenes, with high sequence similarity. We cloned all full-length transcripts from these loci and performed yeast two-hybrid analyses to demonstrate CBL-CIPK interactions and identify specific connections within the network. Using phylogenomics, we have identified three ancient types of CBLs that are discernible by N-terminal localization motifs and a “green algal-type” clade of CIPKs with members from *Physcomitrella* and Arabidopsis.

## Introduction

Of the events that have shaped our modern biosphere, the colonization of land by the predecessors of modern embryophytes stands out as an evolutionary advent that has profoundly affected our landscape and terrestrial ecology. Land plants arose roughly 450 million years ago from a lineage of multicellular freshwater green algae known as charophytes (Graham, 1996; Lewis and McCourt, 2004). Land plants have elaborated and expanded upon a molecular toolkit present in their charophyte ancestors and thereby developed novel anatomical and molecular adaptations to withstand life on land (Graham, 1996; Kenrick and Crane, 1997; Pittermann, 2010; Timme and Delwiche, 2010). The switch from aquatic to terrestrial growth imposed new and formidable abiotic stresses. Discontinuous access to water combined with labile, often unfavorable ion balances spurred the development of sophisticated mechanisms for the perception of water and ion availability, the communication of this information throughout the plant body, and the coordination of orchestrated responses to these stresses.

Calcium ions play a pivotal role in a host of signal transduction cascades in plants as well as in animals. Tightly localized spikes in cytosolic calcium concentration in response to particular environmental cues have been extensively documented in plant cells and are thought to act as early steps in plant signaling pathways (Gilroy et al., 1993; Evans et al., 2001). These bursts, known as calcium signals, are modulated by channels that allow calcium entry from both outside the cell and inside cellular stores (e.g., the vacuole, endoplasmic reticulum). Calcium signals are decoded by proteins that act as sensors; calcium sensors often contain helix-loop-helix motifs known as EF hands that bind calcium and induce conformational changes to modulate the activity of other proteins or domains (Hrabak et al., 2003; McCormack et al., 2005).

Calcineurin B-Like proteins or CBLs are a family of calcium sensors found in all studied land plants and some chlorophyte green algae (Weinl and Kudla, 2009; Batistic et al., 2011). CBLs are named based on their homology to the B regulatory subunit of the phosphatase calcineurin (Luan et al., 2002). CBLs contain four calcium-binding EF hands and typically contain a subcellular localization signal at their N-terminus. The most thoroughly characterized CBLs to date contain a dual lipid modification motif (MGCXXS/T) at their N-terminus that is necessary and sufficient for targeting of fluorescent protein (FP)-fusions to the plasma membrane (Batistic et al., 2008, 2010). Other CBLs are reported to localize to the vacuole, and several of these CBLs contain a distinct N-terminal extension known as the Tonoplast Targeting Sequence (TTS) that targets FP-fusions to the tonoplast (Batistič, 2012; Tang et al., 2012). Uniquely, Arabidopsis CBL10 contains a putative N-terminal transmembrane helix that anchors it to the tonoplast (Kim et al., 2007; Batistic et al., 2010) or plasma membrane (Quan et al., 2007; Ren et al., 2013). Subcellular targeting has been shown to be critical for CBL functionality, and CBLs are responsible for the recruitment and localization of protein partners.

CBLs physically and functionally interact with CBL-Interacting Protein Kinases (CIPKs) and modulate their kinase activity (Shi et al., 1999; Batistic et al., 2011). Hence, the CBL-CIPK network serves to decode calcium signals and transmit these signals through reversible protein phosphorylation. CIPKs, also known as SnRK3 proteins, are serine/threonine protein kinases that consist of a N-terminal kinase domain similar to those found in other plant protein kinases and a unique C-terminal regulatory domain that acts as an autoinhibitory domain and mediates interactions with CBLs. CBLs bind to a short, conserved region within the C-terminal autoinhibitory domain of CIPKs known as the NAF or FISL motif (Shi et al., 1999; Albrecht et al., 2001; Guo et al., 2001). In addition to modulating the kinase activity of CIPKs, CBLs are thought to be the sole or primary determinants of CBL-CIPK complex localization, therefore they are thought to act as functional modules (Luan, 2009; Batistic et al., 2011). CBLs are believed to recruit CIPKs, which lack any sort of discernible targeting signals, to these surfaces, possibly in a calcium-dependent manner (Batističc and Kudla, 2009; Batistic et al., 2010).

Initial functional analysis of the CBL-CIPK network came from the genetic identification of the Salt Overly Sensitive (SOS) pathway. Together, CBL4/SOS3 and CIPK24/SOS2 modulate that activity of the plasma membrane Na^+^/H^+^ exchanger SOS1. Mutants lacking any component of the Salt Overly Sensitive (SOS) pathway display NaCl-hypersensitive phenotypes (Liu and Zhu, 1998; Liu et al., 2000; Shi et al., 2000). CBL4/SOS3 and CIPK24/SOS2 belong to large proteins families containing 10 CBLs and 26 CIPKs in Arabidopsis and similarly sized families in other angiosperms (Kudla et al., 1999; Kolukisaoglu et al., 2004; Weinl and Kudla, 2009). CBL-CIPK complexes have recently been implicated in sodium, potassium, nitrate, and proton transport (Li et al., 2006; Xu et al., 2006; Ho et al., 2009); therefore the CBL-CIPK network is currently thought to be a major regulator of ion homeostasis in angiosperms.

Though CBLs and CIPKs have been discovered among all studied land plants and certain green algal lineages, little is known about the functionality of the CBL-CIPK network outside of angiosperms. As an initial step toward functional analysis of the CBL-CIPK network in an early-diverging land plant, we analyzed the genomic content of CBLs and CIPKs in the model moss *Physcomitrella* and performed bioinformatic analyses of the CBL and CIPK families with an emphasis on relationships among *Physcomitrella* and Arabidopsis CBLs and CIPKs. We classified CBLs according to their phylogeny and N-terminal localization motifs and identified three ancient classes of CBLs. Using yeast two-hybrid analyses, we confirmed interactions among CBLs and CIPKs outside of angiosperms and characterized physical interactions among *Physcomitrella* CBLs and CIPKs. Through phylogenetic analyses, we identified a strongly supported clade that contains all CIPKs identified from green algae and two CIPKs from Arabidopsis and *Physcomitrella*. Using phylogenomic methods, we seek to characterize patterns of expansion of the CBL-CIPK network among land plant lineages to classify CBLs and CIPK in an evolutionarily and functionally meaningful manner to facilitate functional genetic work in early-diverging plants.

## Materials and methods

### Homolog identification, sequence alignment, and bioinformatic analyses

CBL and CIPK homologs were identified using BLASTp and tBLASTn searches of the Uniprot and the NCBI protein and nucleotide databases, using previously identified CBLs and CIPKs from Arabidopsis as queries. Additional sequences were manually retrieved by annotation from UniProt using the keywords “calcineurin” and “CBL-interacting” (Jain et al., 2009). Genomic loci of CBL and CIPK homologs in *Physcomitrella patens* were identified in version 1.6 of the *Physcomitrella* genome, available at http://cosmoss.org (Zimmer et al., 2013). All charophyte CBL and CIPK sequences identified were predicted by assembly of homologous expressed sequence tags (ESTs) from transcriptome-level sequencing of diverse, representative charophyte genera (Timme and Delwiche, 2010; Timme et al., 2012). Other new CBL and CIPK protein sequences were predicted from EST sequences in the NCBI non-redundant (nr) nucleotide database identified by tBLASTn searches. Overlapping ESTs from the same taxa were assembled, and ESTs were translated using Geneious R6 (Biomatters), which was also used for all stages of phylogenetic analyses and figure preparation. Predicted CBL and CIPK homologs were verified by manual inspection of domain architecture and pBLAST searches of the NCBI non-redundant (NR) protein database; all protein sequences included in analyses showed expected domain architecture and yielded top BLASTp hits to previously identified CBLs and CIPKs. CBL and CIPK homologs identified in this study are listed in Supplementary Tables S1,S2, respectively. Protein sequences were aligned using MAFFT (algorithm G-INS-i) and edited and trimmed by eye to remove short, ambiguously aligned regions (see Supplementary Files S1,S2). Edited alignments were used to generate the phylogenetic trees shown Katoh et al. (2002). Phylogenetic trees were generated from the resulting multiple sequence alignments (MSAs) using PhyML with subtree pruning and regrafting (SPR) + nearest neighbor interchange (NNI) moves and *X*^2^-like approximate likelihood ratio test (aLRT) clade support values, which serve as confidence scores much like bootstrap scores. Clades with aLRT scores > 0.95 were deemed to have strong phylogenetic support (Anisimova and Gascuel, 2006; Guindon et al., 2009). Specific model parameters are provided in the figure legend for each PhyML analysis presented, however several additional MSAs and evolutionary models and parameters were tested for agreement with conclusions presented here (data not shown). Clades and evolutionary relationships mentioned in the text appeared consistently in independent phylogenetic analyses with different model parameters and MSAs.

### Cloning and sequencing of CBLs and CIPK from the moss *Physcomitrella*

In order to verify expression and expected splice patterns of CBLs and CIPKs in an early-diverging land plant, we cloned CBLs and CIPKs identified from the model moss *Physcomitrella*. RNA was extracted from protonema and gametophores of *Physcomitrella patens* ssp *patens* ecotype Gransden 2004 using a CTAB/chloroform method similar to the one described by Chang et al. (1993). The RNA was reverse transcribed to produce cDNA using Superscript III Reverse Transcriptase (Invitrogen). Primers containing Invitrogen Gateway attB1 (forward primers) and attB2 (reverse primers) recombination sites were designed to amplify the coding sequences (CDSs) of each *Physcomitrella CBL* (*PpCBL*) and *CIPK* (*PpCIPK*) genes (see Supplementary Table S3 for oligonucleotide sequences used in this study). CBL and CIPK transcripts were amplified using Phusion DNA Polymerase (Thermo-Fisher Scientific) following recommended manufacturer protocols on a MJ Research PTC-100 or PTC-200 model thermocycler. PCR products were visualized on a 0.8% agarose gel, and products of the expected sizes were extracted using a QIAquick gel extraction kit (Qiagen) and cloned into the pDONR™/Zeo vector (Invitrogen) by Gateway BP reaction, following manufacturer recommendations. Samples from three or more clones for each gene were submitted to Elim Biopharmaceuticals, Inc. (Hayward, CA) for DNA sequencing.

### Yeast two-hybrid assays

In order to verify physical interactions among CBLs and CIPKs in a non-angiosperm plant, we cloned the CDS of each full-length *CBL* and *CIPK* transcript identified in *Physcomitrella* and tested interactions among PpCBLs and PpCIPKs in yeast two-hybrid (Y2H) assays using the yeast strain AH109 (Clontech Inc.). This strain is auxotrophic for leucine, tryptophan, histidine, and adenine. The CDSs of *PpCBLs* and *PpCIPKs* were cloned by Gateway LR reaction into yeast two-hybrid gateway-compatible vectors (pGBT9-BS-GW and pGAD-GH-GW) derived from pGBT9-BS and pGAD-GH (Clontech). These vectors were transformed into yeast cells using the G-Biosciences FastYeast Transformation Kit and used to express CBL and CIPK fusions to the DNA-binding domain (BD) and activation domain (AD) of a split transcription factor. We screened CBL-BD fusions (pGBT9-BS-GW constructs) for interactions with CIPK-AD fusion proteins (pGAD-GH-GW constructs) and performed reciprocal screens among CIPK-BD and CBL-AD fusion proteins to verify that the interactions were not vector-dependent. As negative controls, we verified that CBL-BD or CIPK-BD fusion proteins did not interact with the pGAD-GH empty vector (EV).

To perform Y2H screens, co-transformed cells were cultured to mid-log phase in MP Biomedical drop out base (DOB) liquid media lacking leucine and tryptophan (-LT), to ensure retention of vectors containing bait and prey constructs. We then adjusted the cultures to OD_600_ = 0.05 and divided them into four 10-fold dilutions (OD_600_ = 5 × 10^−2^, 5 × 10^−3^, 5 × 10^−4^, 5 × 10^−5^). 6 μl droplets of each dilution were plated on agar DOB media (1) lacking leucine and tryptophan (-LT) to serve as a positive control for transformation and loading, (2) lacking leucine, tryptophan, and histidine (-LTH) to test for protein-protein interactions under low stringency, and (3) lacking leucine, tryptophan, histidine, and adenine (-LTHA) to test for interactions under stringent conditions. Cell growth was recorded at 48 h intervals over the course of 6 days.

## Results and discussion

### CBL-CIPK network composition in green algae, moss, and other land plants

CBLs and CIPKs have been previously identified among various land plants and chlorophyte green algae, though other chlorophytes appear to lack CBL-CIPK homologs (Weinl and Kudla, 2009). Utilizing recently available transcriptome data, we identified CBL and CIPK homologs from several charophyte green algae species: *Coleochaete orbicularis*, *Klebsormidium flaccidum*, *Chaetospheridium globosum*, *Penium margaritaceum*, and *Chlorokybus atmophyticus*. Interestingly, we identified a single CBL and single CIPK in each of these lineages, with one exception. We could not confidently identify a CIPK homolog from *Chlorokybus*, though this may due to incomplete transcriptome coverage. Additional CBL or CIPK homologs may be present in these taxa but undetected due to incomplete sequencing coverage, or additional homologs may simply not be transcribed at sufficient levels under sampled growth conditions. In agreement with our current understanding of evolutionary relationships among these organisms, charophyte green algae sequences display greater sequence similarity to land plant CBLs and CIPKs than chlorophyte homologs. Although there is no currently available genome sequence for any charophyte, only a single CBL and single CIPK were identified in the complete genome sequence of the chlorophytes *Ostreococcus lucimarinus* and *Bathycoccus prasinos*, consistent with prior findings (Weinl and Kudla, 2009). Though it is difficult to make genomic inferences about any charophyte green alga without an available complete genome sequence, our analyses suggest that green algae commonly contain a single CBL-CIPK pair and that the CBL-CIPK network likely predates the split of chlorophyte and charophyte algae.

All CBLs and CIPKs analyzed in this study, including the most divergent homologs identified in algae, show strong conservation of domain architecture and important motifs. At approximately 200 amino acids (AAs) in length, CBLs contain one of a few variations of a localization at their N-termini, followed by 4 calcium-binding EF hand domains. The first EF-hand of CBLs is distinctive in that the calcium-binding loop is comprised of 14 rather than 12 AAs, however evidence suggests that it indeed binds calcium ions (Nagae et al., 2003). Identified full-length CIPKs are approximately 475 AAs in length and have a conserved domain architecture comprised of a N-terminal kinase domain and a C-terminal autoinhibitory region with a diagnostic NAF domain that mediates interactions with CBLs. One previously identified CIPK from the chlorophyte green alga *Chlorella* (UniProt: C4P7Q5) differs, however, in that it possesses 2 NAF domains in its C-terminus, though the significance and accuracy of the published domain architecture is unknown. Our homology search results corroborate the assertion that CBLs and CIPKs are not found in certain chlorophyte green algae, including the models *Chlamydomonas* and *Volvox* (Weinl and Kudla, 2009; Batistic et al., 2011). This pattern parallels trends in calcium channel evolution. The *Chlamydomonas* genome encodes several voltage-dependent calcium channels (VDCCs) and transient receptor potential (TRP) channels, which play critical roles in environmental sensing in metazoans, whereas sequenced land plant genomes do not contain discernible homologs from either family (Wheeler and Brownlee, 2008; Verret et al., 2010). Like most metazoans, *Chlamydomonas* is motile and, in addition to performing photosynthesis, readily grows heterotrophically. *Chlamydomonas* cells contain an organelle unlike any found in plants, the eyespot, that is involved in the calcium-mediated process of phototaxis (Witman, 1993). Based on these observations, it appears that some components of the calcium signaling machinery of certain chlorophyte green algae, such as *Chlamydomonas*, more closely resemble animal signaling networks in some aspects than those of land plants.

**Figure 1:**
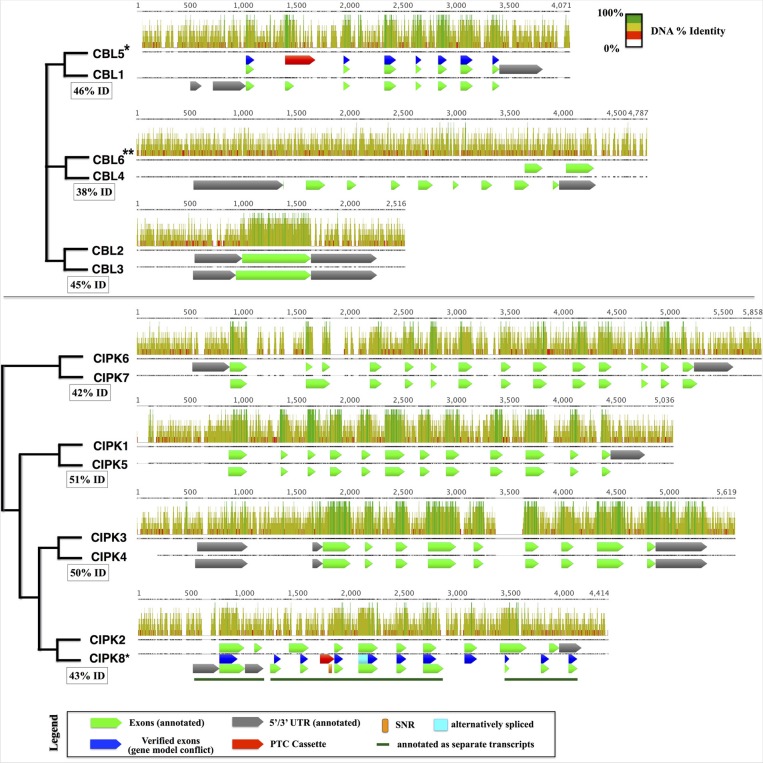
**Pairs of cognate *CBLs* (top) and *CIPKs* (bottom) in the *Physcomitrella* genome aligned using MAFFT**. Displayed pairs of CBLs and CIPKs are genomic loci that are reciprocal best BLASTn hits within the genome, and inferred phylogenetic relationships are indicated by cladograms and described in the main text. Pairwise percentage nucleotide (nt) identity for pairs of genomic loci are displayed in boxes. Aligned nucleotides are displayed as bars shaded proportionally to percentage identity, and gapped regions in the alignment are represented by lines. Bar graphs indicate percentage identity (sliding window = 6 nt). Genes with cloned transcripts that do not encode full-length proteins under tested conditions are indicated with an asterisk (^*^) and genes lacking detectable transcripts are marked with two asterisks (^**^). In cases where our experimentally inferred gene model did not match the annotation, verified exons (blue), alternatively spliced regions (cyan), and premature termination cassettes (PTCs; red) are shown for comparison. *CIPK8*, which was annotated incorrectly as three separate transcripts, contains a long single nucleotide repeat (SNR) comprised of 31 thymidine (T) residues and described further in the main text. Sequences and associated annotations were extracted from the *Physcomitrella* genome v1.6 starting from 500 nucleotides (nt) upstream of the annotated 5′ UTR (750 nt upstream CDS for genes lacking 5′ UTR annotations) to 250 nt downstream of the annotated 5′ UTR (500 nt downstream the CDS) were extracted. Pairwise loci were aligned using MAFFT. Although *PpCBL6* has annotated exons, there is no experimental evidence that any part of this locus in transcribed.

Taking advantage of the published genome sequence of the moss *Physcomitrella patens*, we determined the genomic complement of *CBLs* and *CIPKs* in this early-diverging model plant. We identified a total 4 CBL and 7 CIPK predicted protein sequences in *Physcomitrella*, consistent with prior reports (Batistič and Kudla, 2009; Weinl and Kudla, 2009). One pair of *CBLs* (*PpCBL2*+*3*) and three pairs of *CIPKs* (*PpCIPK1*+*5*, *3*+*4*, and *6*+*7*) showed strikingly high sequence similarities at both the amino acid (73–93% pairwise identity) and genomic level (42–52% pairwise identity). Because of this observation and the inferred whole genome duplication (WGD) estimated to have occurred ~45 million years ago in *Physcomitrella* (Rensing et al., 2007), we hypothesized that pairs of *CBLs* and *CIPKs* are products of the recent WGD and that the “unpaired” *CBLs* (*PpCBL1* and *PpCBL4*) and *CIPK* (*PpCIPK2*) may similarly possess cognate loci in the *Physcomitrella* genome. Consistent with this hypothesis, we identified paired loci for each gene and provisionally named these *PpCBL5*, *PpCBL6*, and *PpCIPK8* (Figure 1). Although these loci showed relatively low percentage identity to their cognate loci compared to previously detected *CBLs* and *CIPKs*, gene predictions using Augustus (Stanke et al., 2004) suggested these loci may encode partial or full-length proteins. Using RT-PCR, we amplified and cloned transcripts from *PpCBL5* and *PpCIPK8*, however we failed to amplify transcripts from the *PpCBL6* locus using several primer pairs validated on genomic DNA (data not shown), despite testing cDNA from different developmental stages (protonema, gametophores, sporophytes). Pairwise alignment of the *PpCBL4* and *PpCBL6* loci revealed a relatively low percentage identity, particularly in *PpCBL4* exonic regions, compared to other “sister” pairs of *CBLs* and *CIPKs*; these observations suggest that *PpCBL6* may be a pseudogene. Sequenced *PpCBL5* and *PpCIPK8* transcripts detected from both gametophyte and sporophyte cDNA were found to contain premature termination cassettes (PTCs) in their spliced forms (see Figure 1, Supplementary File S3), which suggests that these transcripts may not be translated, at least under conditions that we tested. *Physcomitrella CIPK8* contains a single nucleotide repeat (SNR), which are known to promote mutations and quickly change in length (Ellegren, 2004), spanning 31 bases in the retained PTC in cloned transcripts, further marking it as an unusual *CIPK*. Interestingly, *PpCBL5* and *PpCIPK8* show obviously stronger conservation in exonic regions (i.e., regions retained in spliced transcripts and that align to their sister gene's CDS) than intronic or regulatory regions (i.e., promoter and terminator). While these aberrant *CBL* and *CIPK* loci may simply be in early stages of pseudogenization, the unexpected finding that these loci are transcribed and spliced warrants further investigation into possible functions and may point toward a role for these transcripts as regulatory RNAs, as shown previously in animals (Korneev et al., 1999; Hirotsune et al., 2003; Tam et al., 2008). Like in *Physcomitrella*, expansion of the CBL-CIPK network in Arabidopsis, previously attributed to segmental duplications (Kolukisaoglu et al., 2004), can be traced to known WGD events in light of our current understanding of plant genome evolution (Cui et al., 2006). Independent expansion of other gene families in moss and angiosperms has been described, and this can obfuscate direct comparison and functional prediction of genes in widely divergent plants (Cui et al., 2006; Bowman et al., 2007; Rensing et al., 2008; Jiao et al., 2011).

### Phylogenomic analysis of CBL reveals conservation of three unique N-terminal motifs

**Figure 2:**
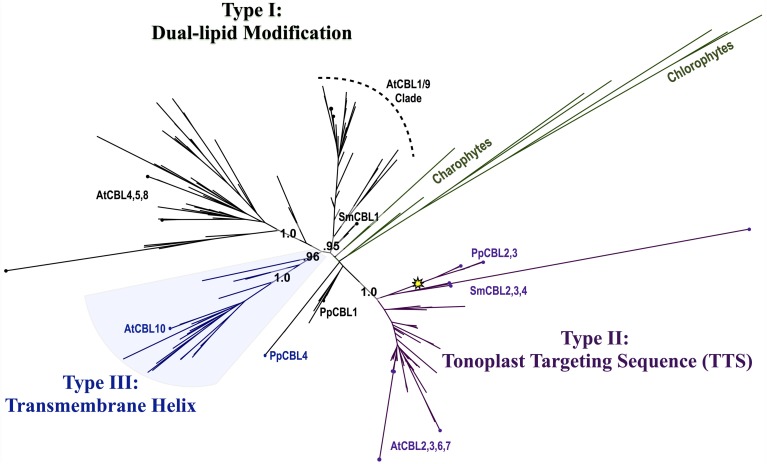
**Maximum Likelihood (ML) phylogenetic tree derived from multiple sequence alignment (MSA) of all CBL amino acid (AA) sequences analyzed in this study**. Chlorophyte and charophyte green algal CBL and CIPKs are highlighted in green, and clades containing Type I–III CBLs identified in this study are annotated. The yellow star indicates an inferred intron loss event. See Supplementary Table S1 for list of CBL sequences used in this study, Supplementary File S1 for MSA, and Supplementary File S2 for full phylogenetic tree.

**Figure 3:**
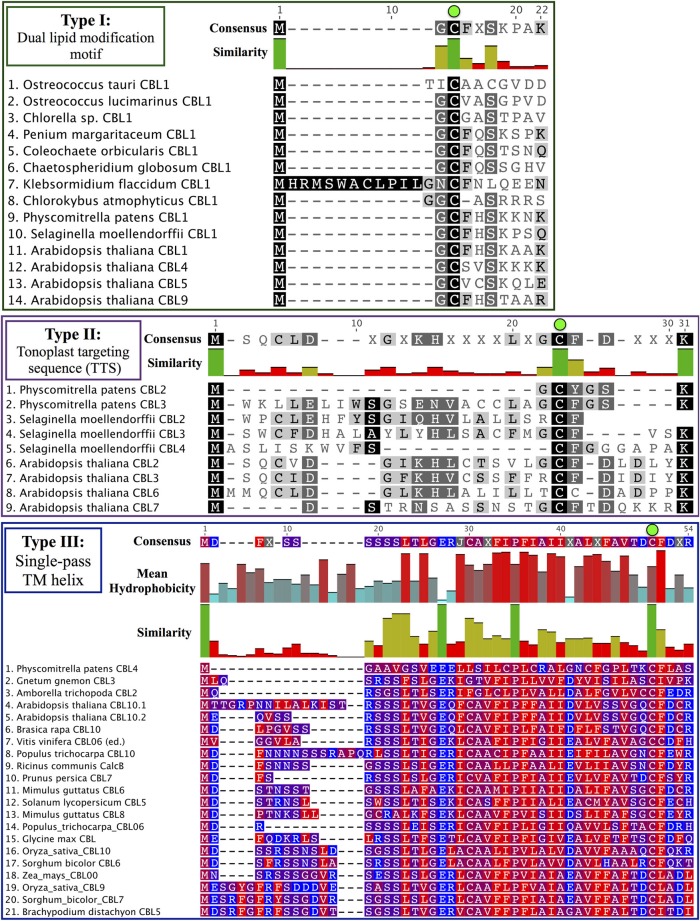
**CBL N-terminal localization motifs can be classified into three ancient types**. Consensus sequences are provided above each MSA, and degree of conservation is indicated by bar graph and shading. Note the strictly conserved cysteine residues (green dots) in all three types of CBLs. (Top) Type I CBLs harbor a dual-lipid modification motif (MGCXXS/T) that triggers N-myristoylation of the glycine residue and S-acylation of the cysteine residue. Most green algal CBLs identified to date are Type I CBLs or appear to retain signatures of the dual-lipid modification motif. (Middle) Type II CBLs are characterized by a N-terminal extension called the TTS that is found in nearly all CBLs contained in the Type II clade. Phylogenetic evidence suggests that PpCBL2 has lost its TTS through a mechanism such as gene conversion. (Bottom) Type III CBLs feature a long N-terminal extension that is predicted to constitute a transmembrane helix. Residues are colored according to hydrophobicity (red) or hydrophilicity (blue), and mean hydrophobicity and similarity are indicated by bar graphs. Although PpCBL4 does not cluster with seed plant CBLs that share a similar N-terminal extension, we propose that it is targeted in a similar manner to other Type III CBLs based on sequence analysis of its N-terminal extension.

Phylogenomic methods extend the ability to determine relationships among distant homologs, facilitate functional prediction, and provide a framework for discovery of key features by identifying conserved regions of proteins (Eisen and Wu, 2002; Sjölander, 2004). Using maximum likelihood (ML) methods, we reconstructed the phylogeny of the CBL family in green lineages. Consistent with our hypothesis that land plant CBLs and CIPKs expanded from a simple module present in their common ancestor with algae, green algal CBLs cluster closely to one another with high confidence scores in phylogenetic analyses. Although algal CBLs do not consistently cluster with any particular clade of CBLs from land plants, they commonly show moderate phylogenetic affinity for a clade containing Arabidopsis CBL1 and CBL9 (Figure 2; see Supplementary Figure 1 for full tree), which play important roles in potassium nutrition through regulation of the AKT1 channel. Like Arabidopsis CBL1 and CBL9, green algal CBLs feature the dual-lipid modification motif MGCXXS/T or obvious relicts of this motif. Due to the retention of this motif among many embryophyte and green algal CBLs and the results of our phylogenetic analysis, we hypothesize that the dual-lipid modification motif is the ancestral localization mechanism of CBLs. This hypothesis is strengthened by the observation that distantly homologous neuronal calcium sensor (NCS) proteins feature a similar N-terminal motif (MGXXXS) that lacks the conserved cysteine residue but does trigger N-myristoylation of the conserved glycine residue (Li et al., 2011). We designate homologs with the dual lipid modification motif as Type I CBLs (Figure 3 *top*). Consistent with the hypothesis that ancestral CBLs most closely resembled modern Type I homologs and gave rise to other types of CBLs, Type I CBLs are paraphyletic with respect to other CBLs. Arabidopsis CBLs containing the Type I dual lipid modification motif have been shown to localize to the plasma membrane (D'Angelo et al., 2006; Cheong et al., 2007; Batistic et al., 2008). Mutational analyses using FP-fusions indicate that both N-myristoylation and S-acylation are required to target proteins to the plasma membrane, whereas either modification on its own results in endomembrane localization (Batistic et al., 2008). Although subcellular localization has not been investigated in early diverging plants or green algae, we speculate that the ancestral CBL-CIPK module may have participated in the regulation of integral membrane proteins at the plasma membrane, given the observed evolutionary trends and our understanding of CBL-CIPK function and biochemistry in Arabidopsis.

**Figure 4:**
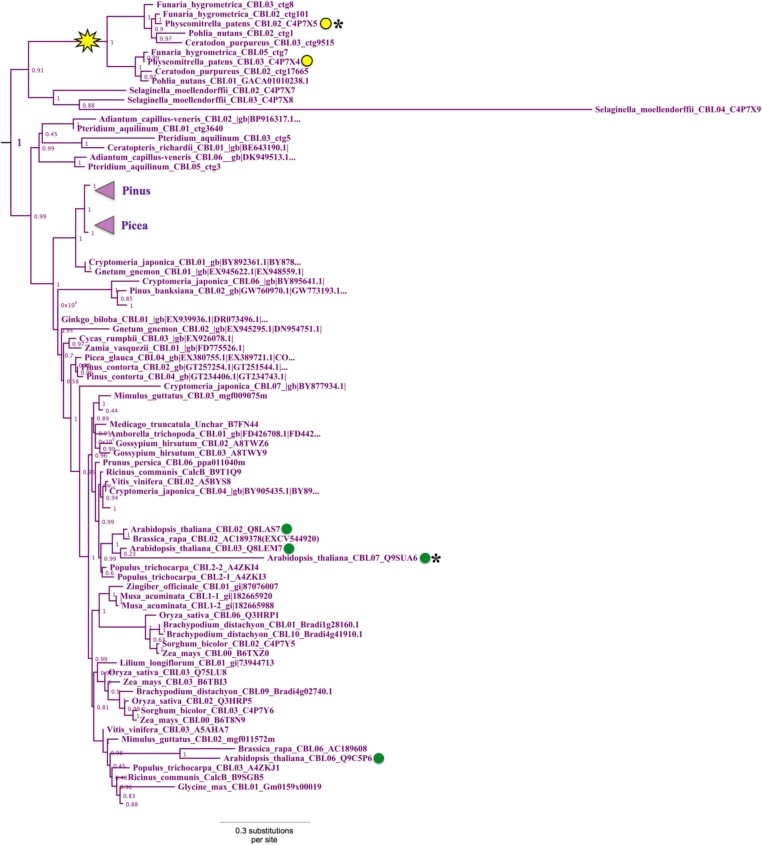
**Close-up of Type II CBL clade from ML tree shown in Figure 2**. Confidence scores (aLRT) are shown for each clade, and the yellow star denotes an inferred intron loss event. Some clades are unlabeled or collapsed for clarity (see full tree in Supplementary File S2). Type II CBLs are distinguished by the presence of an N-terminal tonoplast targeting sequence (TTS), however certain members (asterisks) of the Type II clade have lost or degenerate TTSs. Arabidopsis CBLs in this clade (green dots) contain the TTS or a degenerate form of it; whereas the two *Physcomitrella* CBLs (yellow dots) in this clade sharply differ in that CBL2 has a Type I dual-lipid modification motif and CBL3 has a TTS. Presence of the TTS in *Selaginella* homologs within the clade (see Figure 3) suggests that CBL2 has lost its TTS, through a mechanism such as gene conversion, for example.

Phylogenetic analyses revealed a strongly supported (aLRT = 1.0) clade that contains *Physcomitrella* CBL2 and CBL3 and Arabidopsis CBL2, CBL3, CBL6, and CBL7 (Figure 4). *Physcomitrella CBL2* and *CBL3* encode proteins that are 76% identical, and both genes lack introns, unlike other *CBLs* from Arabidopsis or *Physcomitrella*. The clade also contains homologs from other non-angiosperms, including three CBLs from the lycophyte *Selaginella moellendorffii*. Like Arabidopsis, *Selaginella* CBLs in this clade contain multiple introns and exhibit a conserved exon-intron structure (data not shown), leading us to infer there was a likely reverse transcription event in the *Physcomitrella* lineage not shared with the lineage leading to lycophytes and angiosperms. Experimental work is needed to determine functional consequences of intron loss in *Physcomitrella CBL2* and *CBL3*, however the effects and mechanisms of reverse transcription-mediated intron loss events and other means of intron loss are discussed elsewhere (Jeffares et al., 2006; Filichkin et al., 2010). Based on high sequence similarity, shared intron loss, and strong phylogenetic evidence, we infer that *PpCBL2* and *PpCBL3* are products of a lineage-specific gene duplication, likely the results of a recent WGD (Rensing et al., 2007, 2013). Both genes are orthologous to the four Arabidopsis *CBLs* contained in this clade. Arabidopsis *CBL2* and *CBL3* are also recent duplicates, as evidenced by their phylogenetic placement and very high sequence similarity (~92% AA identity) throughout their entire lengths. *CBL3* and *CBL7* are tandem duplicates, although CBL7 is disparate from other Arabidopsis CBLs and contains a deletion in its N-terminus between a degenerate dual-lipid modification motif and its first EF hand (Batistič and Kudla, 2009). Arabidopsis CBL6, which features an unusual first EF hand relative to other CBLs, is more distantly related to the three other AtCBLs in this clade and forms a clade with orthologous CBLs from other eudicots.

Arabidopsis CBL2, CBL3, and CBL6 have been reported to localize to the tonoplast (Batistič and Kudla, 2009). In the case of CBL2 and CBL3, it has been rigorously shown that an N-terminal motif known as the tonoplast targeting sequence (TTS) mediates its subcellular localization (Tang et al., 2012). The TTSs of Arabidopsis CBL2 and CBL3, with the consensus motif MSQCXDGXKHXCXSXXXCF, span 19 AA; and the last three positions of the motif overlap with positions 2–4 in the dual lipid modification motif of Type I CBLs (i.e., MGCXXS/T), sharing a conserved cysteine residue found in all CBLs analyzed (see Figure 3, Supplementary File S1). This 19-AA fragment from either Arabidopsis CBL2 or CBL3 is necessary and sufficient for targeting of FP fusions to the tonoplast in Arabidopsis mesophyll cells (Tang et al., 2012), and strong sequence similarity suggests that CBL6 shares this targeting mechanism (Figure 3 *middle*). CBL7 is reported to show a diffuse nuclear and cytosolic localization based on the analysis of fluorescent fusion proteins (Batistic et al., 2008), however we are unaware of any rigorous attempts to determine its subcellular localization. Therefore, it appears that tonoplast localization is a generally conserved feature among angiosperm CBLs in this clade. We identified a TTS-like motif in all three *Selaginella* CBLs in this clade and in PpCBL3. Unlike PpCBL3, PpCBL2 does not contain an extended N-terminus and instead contains the Type I dual lipid modification motif. Given these trends, we posit that the TTS is a synapomorphy of this clade and that PpCBL2 lost its TTS via deletion or partial gene conversion, as described elsewhere (Jeffares et al., 2006). Based on strong phylogenetic support and TTS motif conservation, we designate homologs contained in this clade as Type II CBLs.

**Figure 5:**
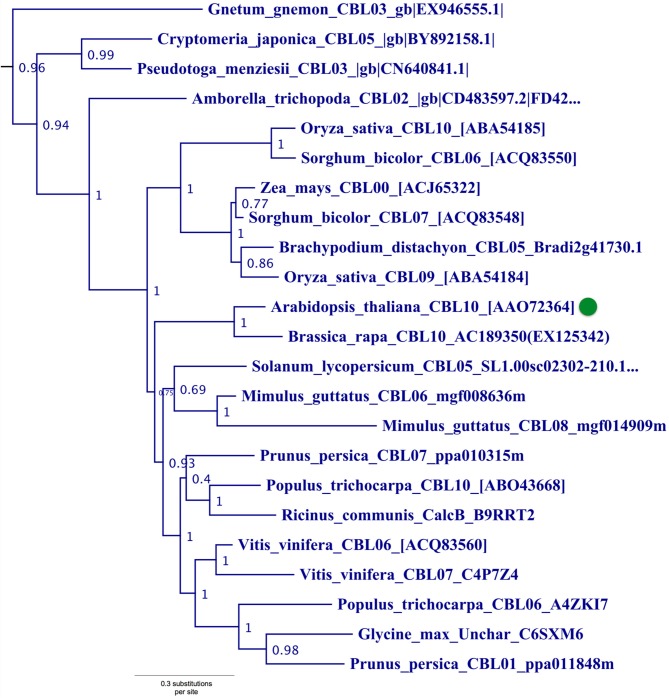
**Close-up of Type III CBL clade from ML tree shown in Figure 2**. Confidence scores (aLRT) are shown for each clade. Arabidopsis CBL10 (green dot) and orthologs in angiosperms and gymnosperms that share the presence of a single N-terminal transmembrane helix (see Figure 3) form a well-supported clade (aLRT = 0.96). *Physcomitrella* CBL4, which also appears to contain a N-terminal transmembrane helix, does not phylogenetically cluster with this clade or other angiosperm homologs, possibly due to sparse taxon sampling among bryophytes.

Phylogenetic analyses also identified a strongly supported clade that contains Arabidopsis CBL10, the only Arabidopsis CBL predicted to contain a transmembrane (TM) helix for membrane association (Figure 5). This clade contains orthologs from all studied angiosperms and gymnosperms, indicating this clade is conserved among seed plants; and all members of this clade with full-length sequences exhibit a predicted N-terminal transmembrane helix. Like members of the AtCBL10 clade, *Physcomitrella* CBL4 contains an extended N-terminus, which we posited may form a transmembrane helix (Figure 3 *bottom*). Various TM topology prediction methods disagree on whether AtCBL10 or PpCBL4 contain a predicted TM helix (data not shown), however visual inspection of hydrophobicity and patterns of conservation in MSAs suggests that both AtCBL10 and PpCBL4 contain N-terminal TM helices. The presence of this TM helix raises the possibility that PpCBL4 may be an AtCBL10 ortholog, however our phylogenetic data neither favor nor disfavor this hypothesis. More thorough coverage of sequence data from early-diverging plants is likely required to test this possibility and determine whether Type III CBLs are monophyletic or not. The Arabidopsis *CBL10* transcript is reportedly processed into mRNAs that encode proteins with two distinct N-termini, though both share the same TM helical region. Alternative splicing is mediated by a unique 8th intron (other rice and Arabidopsis CBLs contain 6 or 7 introns) toward the 5′ pend of the transcript. Both *Physcomitrella CBL4* and Arabidopsis *CBL10* share a very similar exon-intron structure (data not shown), though we did not find evidence of alternative splicing in *PpCBL4*.

The typically short length and strong structural conservation of EF hand proteins like CBLs can complicate phylogenetic reconstruction, as relatively few substitutions can significantly influence results. Due to biophysical constraints, EF hand domains typically exhibit strong sequence conservation at positions that coordinate calcium ion binding. However, variation seen among EF hands of CBLs are predicted to have widely differing affinities for calcium ions, thereby facilitating functional diversity at the level of calcium binding. The 4th EF hand (EF4) of *Physcomitrella* CBL4 is unusual in that it contains non-polar residues at two of the positions that coordinate calcium ion binding, rather than negatively charged residues as seen in virtually all other EF hands. Therefore, it appears likely that it does not bind calcium. Indeed, studies of other calcium signaling pathways have underscored the plasticity of signaling components during evolution. The model yeast *Saccharomyces cerevisiae* contains a single-copy gene encoding a calmodulin (CaM), a widely studied type of calcium sensor in eukaryotes. This gene, *CMD1*, is indispensable for survival of the cell. Surprisingly, molecular genetic analysis suggests the CaM's ability to bind calcium ions is dispensable for its most vital functions, and its fourth EF hand is unable to bind calcium (Cyert, 2001). Plants contain a suite of typical CaMs and widely divergent CaM homologs, some of which either lack the ability to bind calcium ions or coordinate them in an unusual manner (McCormack and Braam, 2003). Further work is needed to clarify the capacity and affinity of identified CBLs for calcium binding, particularly among non-angiosperm CBLs.

There has been some debate as to the localization of Arabidopsis CBL10; various reports indicate localization to the tonoplast (Kim et al., 2007; Batistic et al., 2010) or plasma membrane (Quan et al., 2007; Ren et al., 2013). Although the multiple isoforms of CBL10 may account for different localization patterns, Arabidopsis CBL10 is most strongly expressed in shoots and is suggested to participate in the regulation of a NHX-family, Na^+^/H^+^ exchanger believed to function in the sequestration of sodium ions within the vacuole. A model has emerged wherein CBL10 plays a regulatory role in the SOS pathway akin to that of CBL4/SOS3 (Kim et al., 2007; Tang et al., 2013). In root hair and cortical cells, the Type I Arabidopsis CBL4 forms a complex with CIPK24, and together they regulate the activity of the plasmalemma-localized Na^+^/H^+^ exchanger SOS1 (syn. NHX7) and facilitate the extrusion of sodium ions from the plant. In shoot mesophyll cells, CBL10 complexes with CIPK24, and together they putatively regulate the activity of an unidentified tonoplast-localized Na^+^/H^+^ exchanger and facilitate sequestration of sodium ions in the vacuole. A recent publication proposes a role for CBL10 in the regulation of the plasmalemma-localized potassium channel AKT1 (Ren et al., 2013), which has been rigorously shown to be subject to regulation by CBL1 and CBL9 acting in concert with CIPK23 (Li et al., 2006; Xu et al., 2006). Our phylogenetic results indicate that the single-pass N-terminal TM helix is a synapomorphy of the AtCBL10 clade. *Physcomitrella* CBL4 likewise contains a N-terminal TM helix and may be orthologous, therefore we designated these homologs Type III CBLs.

Different membranes of the eukaryotic cell have distinct phospholipid profiles, which can serve as a basis for subcellular targeting. Moreover, each particular membrane is commonly composed of distinct microenvironments with unique lipid and protein populations. Together, proteins and lipids are thought to form functional modules in cellular membranes, with membrane-targeted kinases recognized as common regulatory modules (Engelman, 2005). For these reasons, we expect that CBL-CIPK complexes are likely targeted not only to specific membranes but to precise sites within membranes where they interact and function with molecular partners (Bhatnagar and Gordon, 1997; Levental et al., 2010). Elevation of free calcium in the cytosol is localized and transient, partly due to effects of Ca^++^-ATPases and Ca^++^/H^+^ antiporters and proteins that act as buffers. Because calcium signatures occur locally, calcium sensors must operate in close proximity to the channels responsible for calcium elevation (Fogelson and Zucker, 1985; Gilroy et al., 1993; Roberts, 1994; Clapham, 2007). In light of this, we interpret the conservation of CBL localization motifs among distantly related plants as a likely consequence of constraints on CBL-CIPK subcellular localization.

Although several studies have examined CBL localization, it remains unclear whether CBLs display a predominantly static or dynamic localization at protein maturity. Our analyses demonstrate that the cysteine residue occupying the third position in the Type I motif (MGCXXS/T) is perfectly conserved among CBLs from widely divergent organisms and paralogous clades. In Type I and Type II CBLs, this residue has been shown to be S-acylated, and the modification is required for known protein functions (Batistic et al., 2008; Batistič, 2012; Tang et al., 2012). Based on its striking conservation, we predict that S-acylation of this conserved residue is a shared among CBLs, at least under certain conditions. It is well established that S-acylation is a reversible post-translational modification and that it can strongly impact protein localization and can be critical for protein function (Bijlmakers and Marsh, 2003; Hemsley and Grierson, 2008). Prior research has pointed toward a role for S-acylation in fine-level targeting of proteins to specific membrane microenvironments (Bhatnagar and Gordon, 1997; Mumby, 1997; Dunphy and Linder, 1998; Levental et al., 2010). We predict that N-terminal S-acylation at this conserved residue functions, at least in part, as a mechanism for precise and dynamic localization of CBLs.

### Conservation of the NAF motif and CBL-CIPK interactions in *Physcomitrella*

**Figure 6:**
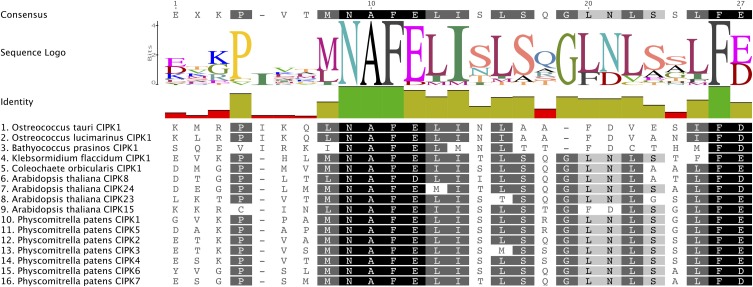
**The CBL-interacting or NAF motif is conserved in green algal and moss CIPKs**. Consensus amino acid sequence of this motif and degree conservation, illustrated by bar graph, are provided above the MSA, which is shaded based on the degree of AA conservation. Strong conservation of this motif, responsible for CIPK interactions with CBLs, suggests that CBLs and CIPKs identified from green algae and early-diverging land plants constitute a functionally linked network.

The CBL-CIPK network is mediated by a conserved CBL-interacting domain (also known as the NAF or FISL motif) in CIPKs. Our MSA of the CIPK family indicates that the NAF domain is strongly conserved, with many identical residues, among algal CIPKs and all CIPKs from Arabidopsis and *Physcomitrella* (Figure 6). This observation is consistent with our prediction that CBLs and CIPKs from green algae and early diverging embryophytes function together as a module. To confirm our presumption that *Physcomitrella* CBLs and CIPKs physically interact with each other and lend support to our interpretation of these protein families as functionally connected in early-diverging plants, we performed Y2H screening and characterized physical interactions between full-length PpCBLs and PpCIPKs in yeast cells.

**Figure 7:**
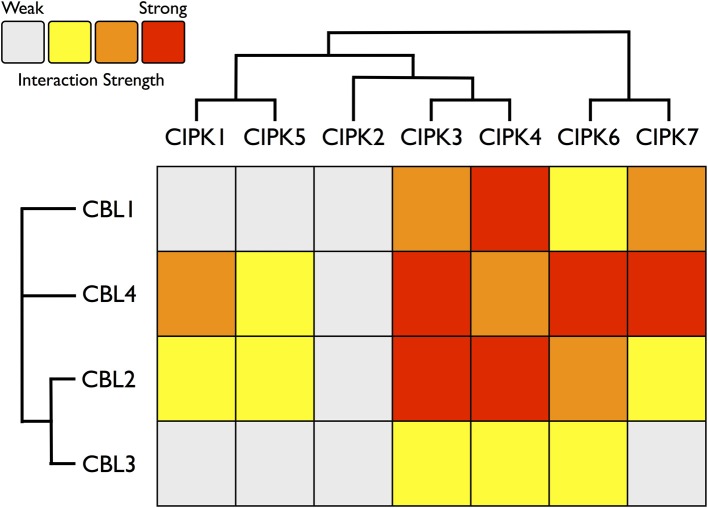
**Heat map summarizing yeast two-hybrid (Y2H) results for all *Physcomitrella* CBL and CIPK combinations**. Each CBL and CIPK was fused to activation domain (AD) or DNA-binding domain (BD) of a split transcription factor and screened for interactions between CBL-AD/CIPK-BD fusion proteins and CBL-BD/CIPK-AD fusions. Interaction strength was inferred by serial growth dilutions on selective media lacking one or two auxotrophic markers and summarized qualitatively by heat map. Red boxes indicate vigorous growth on -LTHA plates (see Materials and Methods); orange boxes indicate weaker growth on -LTHA plates. Yellow boxes indicate robust growth on -LTH plates but no growth on -LTHA plates. Gray boxes indicate weak growth on -LTH plates, but each CBL-CIPK interaction conferred better growth than the empty vector (EV) control. Representative images of each assay are shown in Supplementary Figure S3. Inferred phylogenetic relationships of *Physcomitrella* CBLs and CIPKs are indicated by cladogram and described in the main text.

Consistent with our expectations, CBLs and CIPKs from *Physcomitrella* showed physical interactions in yeast cells. All combinations of PpCBL and PpCIPK fusion proteins showed physical interactions in yeast (Supplementary Figure S2), but specific CBL-CIPK combinations showed very strong interactions with select partners, consistent with the hypothesis that particular CBLs show preferential interactions with cognizant CIPKs (Figure 7). We observed that “creeter” CIPKs displayed overlapping, though not identical, interaction profiles with their most closely related homolog. CIPK1 and CIPK5 interact moderately with CBL2 and CBL4 and weakly with CBL1 and CBL3. CIPK3 and CIPK4 interact weakly with CBL3 but moderately to strongly with CBL1, 2, and 4. CIPK6 and CIPK7 interact strongly with CBL4 and weakly to moderately with CBL1, 2, and 3. We observed only weak interactions between CIPK2, which lacks a “sister” CIPK, and any CBL, despite conservation of its NAF domain and phylogenetic proximity to the highly interactive CIPK3 and CIPK4.

Among the CBLs, CBL4 shows the highest number of strong connections to CIPKs, and it interacts very strongly with CIPK6 and CIPK7, members of the green algal clade of CIPKs. CBL1, a Type I CBL without clear phylogenetic affinities to angiosperm CBLs, most strongly interacts with CIPK4 and shows very weak interactions with CIPK1 and CIPK5. CBL3 shows clearly weaker interactions with CIPKs than its close paralog CBL2, although their interaction profiles are similar. CBL2 and CBL3 both interact most strongly with CIPK3, CIPK4, and CIPK6. These data support the model of a highly interconnected signaling network, however interaction patterns may differ significantly in moss cells due to differences in post-translational modifications, subcellular localization, expression, and other factors. Nonetheless, these results provide a guide for genetic analyses in moss and lend confidence to the interpretation that CBLs and CIPKs are functionally linked in early-diverging plants and constitute an ancient signaling network.

### Phylogenomic identification of the ancestral or “green algal-type” clade of CIPKs

**Figure 8:**
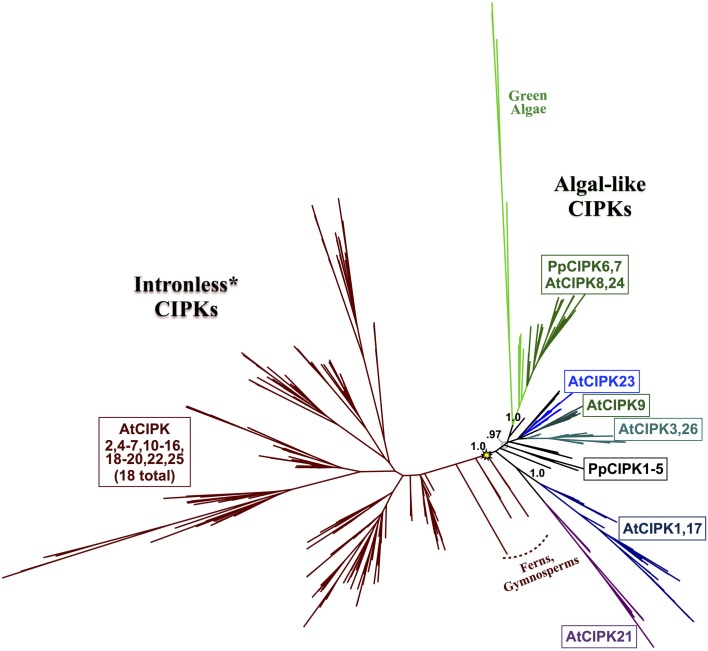
**ML phylogenetic tree derived from protein MSA of all CIPKs identified in this study**. Confidence scores (aLRT) are shown for select clades. CIPKs from green algae phylogenetically cluster with land plant CIPKs, including *Physcomitrella* CIPK6 and CIPK7. Remaining *Physcomitrella* CIPKs cluster with Arabidopsis CIPK1, 17, and 21, which contain multiple introns, and a clade of “intronless” *CIPKs* (although *AtCIPK16* has gained one intron) derived from an inferred reverse transcription event (yellow star). See Supplementary Table S3 for CIPKs in this study, Supplementary File S3 for MSA, and Supplementary File S4 for full phylogenetic tree with tip labels. (^*^Although AtCIPK16 has gained one intron.)

Phylogenomic analyses of the CIPK family were pursued, as described for CBLs, to decipher evolutionary patterns to facilitate identification of functionally meaningful groups, which would be expected to show conservation across diverse land plants. Our phylogenomic analyses of CIPKs (Figure 8; see Supplementary Figure S3 for full tree) indicated most Arabidopsis CIPKs (18 of 26) are contained within an “intronless” clade (although *CIPK16* contains a single intron that is inferred to be from an intron-gain event), consistent with prior analyses by Kolukisaoglu et al. (2004). We used conifer protein sequences from this clade as queries for tBLASTn searches of *Picea* chromosomal sequences (available at http://congenie.org) and did not identify introns in expected locations for intron-containing CIPKs (data not shown). Based on these observations, we posit that a reverse transcription event occurred before the split of gymnosperms and angiosperms and is a conserved feature of this clade. All *Physcomitrella CIPKs* contain multiple introns, and none cluster with the intronless clade. *Physcomitrella* CIPK1—CIPK5 share high sequence similarity (83–93% pairwise); and in our analyses, they were placed with strong confidence in a clade with homologs from other mosses, indicating they are paralogs in respect to their closest seed plant homologs. This clade of moss homologs is likely orthologous (aLRT = 0.97) to three clades of CIPKs conserved across seed plants: the aforementioned intronless clade, a clade containing AtCIPK21, and a clade containing AtCIPK1 and AtCIPK17.

Arabidopsis CIPK3+CIPK26, CIPK9, and CIPK23 each represent strongly supported (aLRT = 1.0; see Supplementary Figure S3) clades that cluster with one another and contain homologs in fully sequenced angiosperm genomes and, at least for the CIPK3 + CIPK26 and CIPK23 clades, in gymnosperms. Whereas CIPK9 and CIPK23 regulate potassium transport and function in root and shoot tissues (Cheong et al., 2007; Pandey et al., 2007), CIPK3 has been implicated in abscisic acid (ABA)-dependent regulation of seed germination (Pandey et al., 2008), therefore homologs from seed plants as distantly related as gymnosperms might conceivably have a conserved regulatory role in seeds, given their strong conservation.

**Figure 9:**
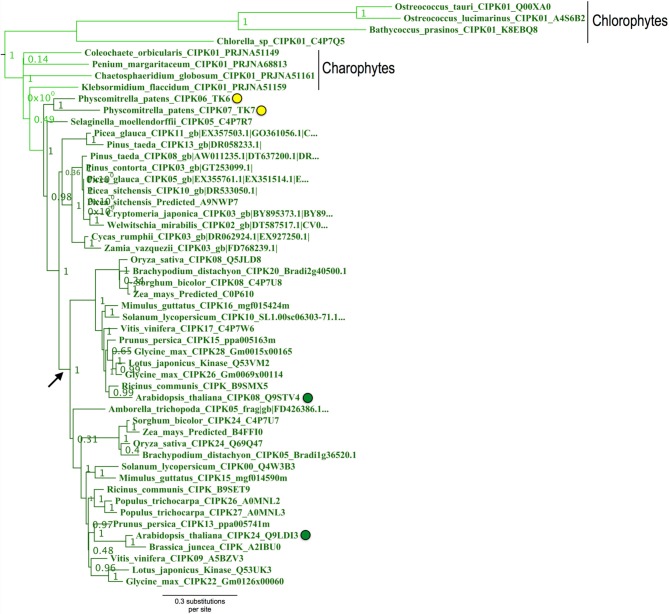
**Close-up of the “green algal-type” CIPK clade from ML tree shown in Figure 8**. Confidence scores (aLRT) are shown for each clade. Phylogenetic evidence strongly supports the existence of a clade (aLRT = 1.0) containing all CIPK homologs identified from chlorophyte and charophyte green algae, as well as two CIPKs each from *Physcomitrella* (yellow dots) and Arabidopsis (green dots). *Physcomitrella* CIPK6 and CIPK7 are recent paralogs and sister to one another in our analyses. In contrast, Arabidopsis CIPK8 and CIPK24 each have clear orthologs in other sequenced angiosperms, and these clades appear to have arisen from a gene duplication that occurred around the time of divergence of angiosperms (arrow).

*Physcomitrella* CIPK6 and CIPK7 belong to a clade that contains Arabidopsis CIPK8 and CIPK24 and, importantly, contains all green algal CIPKs identified (Figure 9) with high confidence (aLRT = 1.0). Although *Physcomitrella* and Arabidopsis each contain two homologs in this clade, *Physcomitrella* CIPK6 and CIPK7 (72% AA pairwise identity) are the products of a gene duplication that occurred after the split between mosses and the lineage leading to vascular plants. In contrast, Arabidopsis CIPK8 and CIPK24 (60% pairwise identity) each represent a separate, strongly supported clade with orthologs in other angiosperms, implying that they derive from duplications that occurred during seed plant (most likely angiosperm) diversification. Based on our results, we posit that *Physcomitrella* CIPK6 and CIPK7 and Arabidopsis CIPK8 and CIPK24 (SOS2) most closely resemble the ancestral or “green algal-type” CIPK and, due to their orthology, most likely to reflect ancestral function(s) of the CBL-CIPK network.

**Figure 10:**
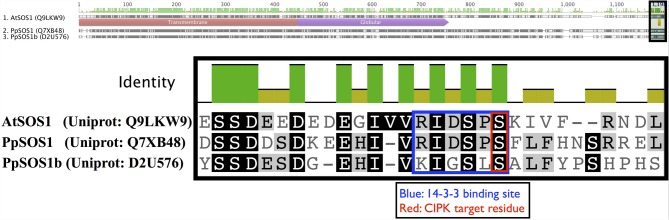
**Key regulatory residues at the C-terminus of Arabidopsis SOS1 (AtSOS1) are conserved in *Physcomitrella* homologs**. The full-length protein sequence alignment is shown on top with the zoomed in region indicated by a shaded box. Arabidopsis CIPK24 (SOS2) phosphorylates the serine (S) residue marked in red in AtSOS1 and facilitates binding by a 14-3-3 protein. In Arabidopsis, these mechanisms are critical to plant sodium tolerance. Strong sequence conservation suggest similar mechanisms may be in place in *Physcomitrella*, though the cognate CBL-CIPK pair is currently unknown.

Arabidopsis CIPK8 is believed to be a positive regulator of the low-affinity phase of the primary nitrate response and has been implicated in glucose sensing, although mechanistic details are unknown at this time (Hu et al., 2009). Arabidopsis CIPK24, the first functionally characterized CIPK, plays a critical function in sodium tolerance through CBL4(SOS3)-modulated phosphorylation of the Na^+^/H^+^ exchanger SOS1. There is substantial evidence that orthologs of CBL4 and CIPK24 in other flowering plant lineages have similar functions (Martínez-Atienza et al., 2007; Tang et al., 2010). Given the phylogenetic proximity of Arabidopsis CIPK24 to green algal CIPKs, future work will test whether green algal CIPKs, and *Physcomitrella* CIPK6 and CIPK7, function in Na^+^/K^+^ homeostasis or possibly more broadly regulate ion transport. It has already been established that two orthologs of SOS1 in *Physcomitrella* (PpSOS1 and PpSOS1b) are required for proper K^+^/Na^+^ ratios and sodium tolerance (Quintero et al., 2011). Interestingly, a 6 AA C-terminal motif of AtSOS1 that is a phosphorylation substrate of CIPK24 and a 14-3-3 protein-binding site is 100% identical to PpSOS1 and 50% identical to PpSOS1b, and the target serine is conserved in both homologs (Figure 10). *Physcomitrella* SOS1 has further been shown to confer enhanced NaCl tolerance when heterologously expressed in yeast, and the effect is strengthened by coexpression with Arabidopsis CBL4 and CIPK24 (Fraile-Escanciano et al., 2010). Collectively, these observations suggest that the SOS pathway is conserved across land plants and may be conserved among some green algal lineages. Functional molecular analyses of CBLs and CIPKs in early-diverging plant and algal lineages could provide core insights and clarify the increasingly complex picture of calcium-regulated abiotic stress responses in Arabidopsis and agricultural species.

## Conclusions

Prior publications (e.g., Batistič and Kudla, 2009; Weinl and Kudla, 2009) have mentioned the apparent expansion of the CBL-CIPK network in terms of the total numbers of CBLs and CIPKs found in algae and early diverging plants compared to their angiosperm counterparts. Here, we present phylogenetic evidence that the CBL-CIPK network has expanded independently in multiple plant lineages, including mosses and angiosperms. It appears that the common ancestor of mosses and vascular plants likely contained three CBLs distinguishable by N-terminal localization motifs, which likely are synapomorphies among ancient CBL subfamilies. We have identified a clade of CIPKs containing all green algal homologs and two representatives from *Physcomitrella* and Arabidopsis. Phylogenetic analysis demonstrates that the *Physcomitrella* and Arabidopsis members of this clade are the products of independent gene duplications and the earliest land plants likely contained a single homolog from this clade. The concurrent pairing of *CBLs* and *CIPKs* in available genomes and transcriptomes, the striking conservation of the NAF domain, and our Y2H results all point toward a physically and functionally connected CBL-CIPK network across plants and algae.

The function(s) of CBL-CIPK pairs found in green algae remains an open and intriguing question, and our identification of charophyte CBL-CIPK pairs expands the list of potential models for this inquiry. The conspicuous expansion of the network in several land plant lineages appears to have been driven largely by WGDs, and we hypothesize that duplicated members were adapted for novel signaling pathways and precise roles in particular cells and tissues. Research on molecular processes modulated by CBLs and CIPKs has intensified in recent years, and researchers are beginning to investigate CBL-CIPK functions in non-model angiosperm species. The field is prime for investigation of CBL-CIPK functions in earlier diverging land plants, and research in this area will enhance our understanding of the molecular evolutionary basis of the colonization of land by plants.

## Funding

This research is supported by a grant from the National Science Foundation (to Sheng Luan).

### Conflict of interest statement

The authors declare that the research was conducted in the absence of any commercial or financial relationships that could be construed as a potential conflict of interest.

## Supplementary material

Supplementary Table S1 **CBL sequences used in this study**.

Supplementary Table S2 **CIPK sequences used in this study**.

Supplementary Table S3 **Oligonucleotides used in this study**.
