# Supplementary figures and images for: Comparative phylogenomics of the CBL-CIPK calcium-decoding network in the moss Physcomitrella, Arabidopsis, and other green lineages
Source: Front Plant Sci. 2014 May 14;5:187. doi: 10.3389/fpls.2014.00187 (PMC4030171; doi:10.3389/fpls.2014.00187)

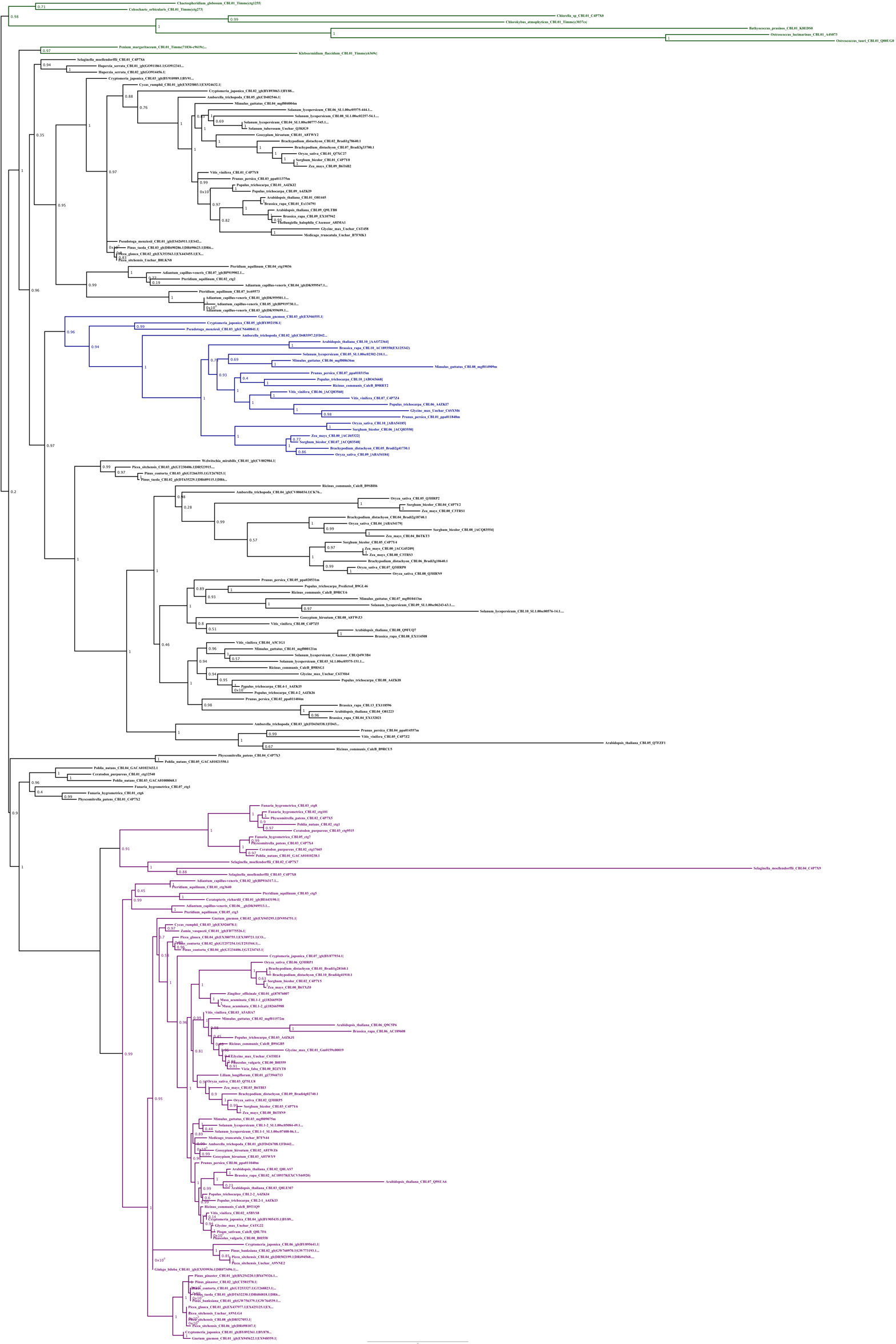

Supplement: Supplementary file 2 [file Presentation1.PDF]
