# Supplementary material for: Comparative phylogenomics of the CBL-CIPK calcium-decoding network in the moss Physcomitrella, Arabidopsis, and other green lineages
Source: Front Plant Sci. 2014 May 14;5:187. doi: 10.3389/fpls.2014.00187 (PMC4030171; doi:10.3389/fpls.2014.00187)
Supplement: Supplementary Table S1 — CBL sequences used in this study. [file DataSheet4.PDF]

Species	Genus	Description	ID / Accession	Source	Sequence (residues included in MSA)
Adiantum	capillus-veneris	CBL01	gb|DK959501.1|DK960240.1|DK957557.1|BP921233.1|	NCBI GenBank	[illegible]
Adiantum	capillus-veneris	CBL02	gb|BP916317.1|BP914025.1|	NCBI GenBank	[illegible]
Adiantum	capillus-veneris	CBL03	gb|DK959699.1|	NCBI GenBank	[illegible]
Adiantum	capillus-veneris	CBL04	gb|DK959547.1|	NCBI GenBank	[illegible]
Adiantum	capillus-veneris	CBL05	gb|BP919730.1|	NCBI GenBank	[illegible]
Adiantum	capillus-veneris	CBL06 frag	gb|DK949513.1|	NCBI GenBank	[illegible]
Adiantum	capillus-veneris	CBL07	gb|BP919902.1|	NCBI GenBank	[illegible]
Amborella	trichopoda	CBL01	gb|FD426708.1|FD442481.1|FD433735.1|FD426697.1|FD431456.1|	NCBI GenBank	[illegible]
Amborella	trichopoda	CBL02	gb|CD483597.2|FD428407.1|FD429741.1|CK753784.1|CD482054.2|	NCBI GenBank	[illegible]
Amborella	trichopoda	CBL03	gb|FD436538.1|FD436453.1|	NCBI GenBank	[illegible]
Amborella	trichopoda	CBL04	gb|CV006034.1|CK762933.1|	NCBI GenBank	[illegible]
Amborella	trichopoda	CBL05	gb|CD482546.1|	NCBI GenBank	[illegible]
Arabidopsis	thaliana	CBL01	O81445	UniProt	[illegible]
Arabidopsis	thaliana	CBL02	Q8LAS7	UniProt	[illegible]
Arabidopsis	thaliana	CBL03	Q8LEM7	UniProt	[illegible]
Arabidopsis	thaliana	CBL04	O81223	UniProt	[illegible]
Arabidopsis	thaliana	CBL05	Q7FZF1	UniProt	[illegible]
Arabidopsis	thaliana	CBL06	Q9C5P6	UniProt	[illegible]
Arabidopsis	thaliana	CBL07	Q9SUA6	UniProt	[illegible]
Arabidopsis	thaliana	CBL08	Q9FUQ7	UniProt	[illegible]
Arabidopsis	thaliana	CBL09	Q9LTB8	UniProt	[illegible]
Arabidopsis	thaliana	CBL10 (CBL10.1)	AAO72364	NCBI GenBank	[illegible]
Arabidopsis	thaliana	CBL10 (CBL10.2)	Q7FRS8-2	UniProt	[none in published phylogenetic tress , see AtCBL10.1]
Bathyococcus	prasinos	CBL01	K8EDS0	UniProt	[illegible]
Brachypodium	distachyon	CBL01	Bradi1g28160.1	Brachypodium.org	[illegible]
Brachypodium	distachyon	CBL02	Bradi1g78640.1	Brachypodium.org	[illegible]
Brachypodium	distachyon	CBL04	Bradi2g18740.1	Brachypodium.org	[illegible]
Brachypodium	distachyon	CBL05	Bradi2g41730.1	Brachypodium.org	[illegible]
Brachypodium	distachyon	CBL06	Bradi3g10640.1	Brachypodium.org	[illegible]
Brachypodium	distachyon	CBL07	Bradi3g33700.1	Brachypodium.org	[illegible]
Brachypodium	distachyon	CBL09	Bradi4g02740.1	Brachypodium.org	[illegible]
Brachypodium	distachyon	CBL10	Bradi4g41910.1	Brachypodium.org	[illegible]
Brassica	rapa	CBL01	Ex134791	NCBI GenBank	[illegible]
Brassica	rapa	CBL02	AC189378(EXCV544920)	NCBI GenBank	[illegible]
Brassica	rapa	CBL04	EX132021	NCBI GenBank	[illegible]
Brassica	rapa	CBL06	AC189608	NCBI GenBank	[illegible]
Brassica	rapa	CBL08	EX114508	NCBI GenBank	[illegible]
Brassica	rapa	CBL09	EX107942	NCBI GenBank	[illegible]
Brassica	rapa	CBL10	AC189350(EX125342)	NCBI GenBank	[illegible]
Brassica	rapa	CBL13	EX118596	NCBI GenBank	[illegible]
Ceratodon	purpureus	CBL01	contig12540 (PRJNA79853)	NCBI BioProject	[illegible]
Ceratodon	purpureus	CBL02	contig17665 (PRJNA79853)	NCBI BioProject	[illegible]
Ceratodon	purpureus	CBL03	contig9515 (PRJNA79853)	NCBI BioProject	[illegible]
Ceratopteris	richardii	CBL01	gb|BE643190.1|	NCBI GenBank	[illegible]
Chaetosphaeridium	globosum	CBL01	Timme|Contig1255 (PRJNA51161)	NCBI BioProject	[illegible]
Chlorella	sp	CBL01	C4P7X0	UniProt	[illegible]
Chlorokybus	atmophyticus	CBL01	Timme|c3037cs| (PRJNA51157)	NCBI BioProject	[illegible]
Coleochaete	orbicularis	CBL01	Timme|Contig273| (PRJNA51149)	NCBI BioProject	[illegible]
Cryptomeria	japonica	CBL01	gb|BY892361.1|BY878579.1|BY888108.1|BY881209.1|BY894587.1|BY883105.1|BY880687.1|BY878225.1|[illegible]	NCBI GenBank	[illegible]
Cryptomeria	japonica	CBL02	gb|BY893063.1|BY886332.1|	NCBI GenBank	[illegible]
Cryptomeria	japonica	CBL03	gb|BY910989.1|BY910554.1|	NCBI GenBank	[illegible]
Cryptomeria	japonica	CBL04	gb|BY905435.1|BY896817.1|	NCBI GenBank	[illegible]
Cryptomeria	japonica	CBL05	gb|BY892158.1|	NCBI GenBank	[illegible]
Cryptomeria	japonica	CBL06	gb|BY895641.1|	NCBI GenBank	[illegible]
Cryptomeria	japonica	CBL07	gb|BY877934.1|	NCBI GenBank	[illegible]
Cycas	rumphii	CBL01	gb|EX925803.1|EX924632.1|	NCBI GenBank	[illegible]
Cycas	rumphii	CBL03	gb|EX926078.1|	NCBI GenBank	[illegible]
Funaria	hygrometrica	CBL01	contig6	P.C. Peter Szovenyi (University of Zurich)	[illegible]
Funaria	hygrometrica	CBL02	contig101	P.C. Peter Szovenyi (University of Zurich)	[illegible]
Funaria	hygrometrica	CBL03	contig8	P.C. Peter Szovenyi (University of Zurich)	[illegible]
Funaria	hygrometrica	CBL05	contig7	P.C. Peter Szovenyi (University of Zurich)	[illegible]
Funaria	hygrometrica	CBL07	contig1	P.C. Peter Szovenyi (University of Zurich)	[illegible]
Ginkgo	biloba	CBL01	gb|EX939936.1|DR073496.1|DR074511.1|	NCBI GenBank	[illegible]
Glycine	max	CBL01	Gm0159x00019 (K7LBA3)	UniProt	[illegible]
Glycine	max	Unchar	C6SXM6	UniProt	[illegible]
Glycine	max	Unchar	C6T458	UniProt	[illegible]
Glycine	max	Unchar	C6TG22	UniProt	[illegible]
Glycine	max	Unchar	C6THE4	UniProt	[illegible]
Glycine	max	Unchar	C6TM64	UniProt	[illegible]
Gnetum	gnemon	CBL01	gb|EX945622.1|EX948559.1|	NCBI GenBank	[illegible]
Gnetum	gnemon	CBL02	gb|EX945295.1|DN954751.1|	NCBI GenBank	[illegible]
Gnetum	gnemon	CBL03	gb|EX946555.1|	NCBI GenBank	[illegible]
Gossypium	hirsutum	CBL01	A8TWY2	UniProt	[illegible]
Gossypium	hirsutum	CBL02	A8TWZ6	UniProt	[illegible]
Gossypium	hirsutum	CBL03	A8TWY9	UniProt	[illegible]
Gossypium	hirsutum	CBL08	A8TWZ3	UniProt	[illegible]
Huperzia	serrata	CBL01	gb|GO911861.1|GO912341.1|	NCBI GenBank	[illegible]
Huperzia	serrata	CBL02	gb|GO914456.1|	NCBI GenBank	[illegible]
Klebsormidium	flaccidum	CBL01	Timme|c6369c| (PRJNA51159)	NCBI BioProject	[illegible]
Lilium	longiflorum	CBL01	gi|73944713	NCBI GenBank	[illegible]
Medicago	truncatula	Unchar	B7FMK1	UniProt	[illegible]
Medicago	truncatula	Unchar	B7FN44	UniProt	[illegible]
Mimulus	guttatus	CBL01	mgf000121m	Phytozome	[illegible]
Mimulus	guttatus	CBL02	mgf011572m	Phytozome	[illegible]
Mimulus	guttatus	CBL03	mgf009075m	Phytozome	[illegible]
Mimulus	guttatus	CBL04	mgf004004m	Phytozome	[illegible]
Mimulus	guttatus	CBL06	mgf008636m	Phytozome	[illegible]
Mimulus	guttatus	CBL07	mgf010413m	Phytozome	[illegible]
Mimulus	guttatus	CBL08	mgf014909m	Phytozome	[illegible]
Musa	acuminata	CBL1-1	gi|182665920	NCBI GenBank	[illegible]
Musa	acuminata	CBL1-2	gi|182665988	NCBI GenBank	[illegible]
Oryza	sativa	CBL01	Q7XC27	UniProt	[illegible]
Oryza	sativa	CBL02	Q3HRP5	UniProt	[illegible]
Oryza	sativa	CBL03	Q75LU8	UniProt	[illegible]
Oryza	sativa	CBL04	ABA54179	NCBI GenBank	[illegible]
Oryza	sativa	CBL05	Q3HRP2	UniProt	[illegible]
Oryza	sativa	CBL06	Q3HRP1	UniProt	[illegible]
Oryza	sativa	CBL07	Q3HRP0	UniProt	[illegible]
Oryza	sativa	CBL08	Q3HRN9	UniProt	[illegible]
Oryza	sativa	CBL09	ABA54184	NCBI GenBank	[illegible]
Oryza	sativa	CBL10	ABA54185	NCBI GenBank	[illegible]
Ostreococcus	lucimarinus	CBL01	A4S873	UniProt	[illegible]
Ostreococcus	tauri	CBL01	Q00UG0	UniProt	[illegible]
Penium	margaritaceum	CBL01	Timme|71836-c9619c|71836-c35309c|71836-c19386c| (PRJNA68813)	NCBI BioProject	[illegible]
Phaseolus	vulgaris	CBL00	B0I558	UniProt	[illegible]
Phaseolus	vulgaris	CBL00	B0I559	UniProt	[illegible]
Physcomitrella	patens	CBL01	C4P7X2	UniProt	[illegible]
Physcomitrella	patens	CBL02	C4P7X5	UniProt	[illegible]
Physcomitrella	patens	CBL03	C4P7X4	UniProt	[illegible]
Physcomitrella	patens	CBL04	C4P7X3	UniProt	[illegible]
Picea	glauca	CBL01	gb|EX437977.1|EX425125.1|EX422785.1|GE473595.1|DV993265.1|DV970594.2|EX438720.1|EX327621.1|[illegible]	NCBI GenBank	[illegible]
Picea	glauca	CBL02	gb|EX353563.1|EX443455.1|EX420421.1|EX353604.1|EX405578.1|DR568819.1|DR568323.1|EX413838.1|[illegible]	NCBI GenBank	[illegible]
Picea	glauca	CBL04	gb|EX380755.1|EX389721.1|CO255542.1|	NCBI GenBank	[illegible]
Picea	sitchensis	CBL03	gb|GT230406.1|DR523915.1|DR533779.1|GT125998.1|GT123836.1|DR515718.1|ES248948.1|CO226630.1|	NCBI GenBank	[illegible]
Picea	sitchensis	CBL04	gb|DR502199.1|DR494568.1|	NCBI GenBank	[illegible]
Picea	sitchensis	CBL06	gb|DR498107.1|	NCBI GenBank	[illegible]
Picea	sitchensis	CBL08	gb|DR527053.1|	NCBI GenBank	[illegible]
Picea	sitchensis	Unchar	A9NLG4	UniProt	[illegible]
Picea	sitchensis	Unchar	A9NNE2	UniProt	[illegible]
Picea	sitchensis	Unchar	B8LKN8	UniProt	[illegible]
Pinus	banksiana	CBL01	gb|GW756379.1|GW764539.1|GW753015.1|GW744741.1|GW767366.1|GW760431.1|GW745072.1|GW7533[illegible]	NCBI GenBank	[illegible]
Pinus	banksiana	CBL02	gb|GW760970.1|GW773193.1|GW761307.1|GW773548.1|	NCBI GenBank	[illegible]
Pinus	contorta	CBL01	gb|GT253327.1|GT268823.1|GT249925.1|GT238176.1|GT269452.1|	NCBI GenBank	[illegible]
Pinus	contorta	CBL02	gb|GT257254.1|GT251544.1|GT251916.1|GT257608.1|	NCBI GenBank	[illegible]
Pinus	contorta	CBL03	gb|GT266355.1|GT267025.1|	NCBI GenBank	[illegible]
Pinus	contorta	CBL04	gb|GT234406.1|GT234743.1|	NCBI GenBank	[illegible]
Pinus	pinaster	CBL01	gb|BX254220.1|BX679326.1|	NCBI GenBank	[illegible]
Pinus	pinaster	CBL02	gb|CT581570.1|	NCBI GenBank	[illegible]
Pinus	taeda	CBL01	gb|DT632230.1|DR684818.1|DR684074.1|CX646085.1|CO364900.1|DT632284.1|DR684541.1|DR168621.1|[illegible]	NCBI GenBank	[illegible]
Pinus	taeda	CBL02	gb|DT635229.1|DR689115.1|DR681831.1|DR693578.1|DR080712.1|	NCBI GenBank	[illegible]
Pinus	taeda	CBL03	gb|DR690286.1|DR690623.1|DR688300.1|DR079699.1|	NCBI GenBank	[illegible]
Pisum	sativum	CalcB	Q8L7F6	UniProt	[illegible]
Pohlia	nutans	CBL01	GACA01010238.1	NCBI GenBank	[illegible]
Pohlia	nutans	CBL02	contig1 (GACA01028842.1|GACA01003847.1)	NCBI GenBank	[illegible]
Pohlia	nutans	CBL03	GACA01008068.1	NCBI GenBank	[illegible]
Pohlia	nutans	CBL04	GACA01023432.1	NCBI GenBank	[illegible]
Pohlia	nutans	CBL05	GACA01021550.1	NCBI GenBank	[illegible]
Populus	trichocarpa	CBL01	A4ZKI2	UniProt	[illegible]
Populus	trichocarpa	CBL03	A4ZKJ1	UniProt	[illegible]
Populus	trichocarpa	CBL06	A4ZKI7	UniProt	[illegible]
Populus	trichocarpa	CBL08	A4ZKI8	UniProt	[illegible]
Populus	trichocarpa	CBL09	A4ZKI9	UniProt	[illegible]
Populus	trichocarpa	CBL10	ABO43668	NCBI GenBank	[illegible]
Populus	trichocarpa	CBL2-1	A4ZKI3	UniProt	[illegible]
Populus	trichocarpa	CBL2-2	A4ZKI4	UniProt	[illegible]
Populus	trichocarpa	CBL4-1	A4ZKI5	UniProt	[illegible]
Populus	trichocarpa	CBL4-2	A4ZKI6	UniProt	[illegible]
Populus	trichocarpa	Predicted	B9GL46	UniProt	[illegible]
Prunus	persica	CBL01	ppa011848m	Rosaceae.org	[illegible]
Prunus	persica	CBL02	ppa011404m	Rosaceae.org	[illegible]
Prunus	persica	CBL03	ppa011375m	Rosaceae.org	[illegible]
Prunus	persica	CBL04	ppa014557m	Rosaceae.org	[illegible]
Prunus	persica	CBL05	ppa020531m	Rosaceae.org	[illegible]
Prunus	persica	CBL06	ppa011040m	Rosaceae.org	[illegible]
Prunus	persica	CBL07	ppa010315m	Rosaceae.org	[illegible]
Pseudotoga	menziesii	CBL01	gb|ES426911.1|ES426602.1|ES424379.1|	NCBI GenBank	[illegible]
Pseudotoga	menziesii	CBL03	gb|CN640841.1|	NCBI GenBank	[illegible]
Pteridium	aquilinum	CBL01	contig3640 (PRJNA48473)	NCBI BioProject	[illegible]
Pteridium	aquilinum	CBL02	contig2 (PRJNA48473)	NCBI BioProject	[illegible]
Pteridium	aquilinum	CBL03	contig5 (PRJNA48473)	NCBI BioProject	[illegible]
Pteridium	aquilinum	CBL04	contig19036 (PRJNA48473)	NCBI BioProject	[illegible]
Pteridium	aquilinum	CBL05	contig3 (PRJNA48473)	NCBI BioProject	[illegible]
Pteridium	aquilinum	CBL07	lrc69573 (PRJNA48473)	NCBI BioProject	[illegible]
Ricinus	communis	CalcB	B9RCU5	UniProt	[illegible]
Ricinus	communis	CalcB	B9RCU6	UniProt	[illegible]
Ricinus	communis	CalcB	B9RRT2	UniProt	[illegible]
Ricinus	communis	CalcB	B9RSG1	UniProt	[illegible]
Ricinus	communis	CalcB	B9SBH6	UniProt	[illegible]
Ricinus	communis	CalcB	B9SGB5	UniProt	[illegible]
Ricinus	communis	CalcB	B9T1Q9	UniProt	[illegible]
Selaginella	moellendorffii	CBL01	C4P7X6	UniProt	[illegible]
Selaginella	moellendorffii	CBL02	C4P7X7	UniProt	[illegible]
Selaginella	moellendorffii	CBL03	C4P7X8	UniProt	[illegible]
Selaginella	moellendorffii	CBL04	C4P7X9	UniProt	[illegible]
Solanum	lycopersicum	CAsensor	CBLQ4W3B4	NCBI GenBank	[illegible]
Solanum	lycopersicum	CBL03	SL1.00sc05575-151.1.1	Sol Genomics Network	[illegible]
Solanum	lycopersicum	CBL04	SL1.00sc00777-545.1.1	Sol Genomics Network	[illegible]
Solanum	lycopersicum	CBL05	SL1.00sc02302-210.1.1	Sol Genomics Network	[illegible]
Solanum	lycopersicum	CBL06	SL1.00sc05575-444.1.1	Sol Genomics Network	[illegible]
Solanum	lycopersicum	CBL08	SL1.00sc02257-54.1.1	Sol Genomics Network	[illegible]
Solanum	lycopersicum	CBL09	SL1.00sc06243-63.1.1	Sol Genomics Network	[illegible]
Solanum	lycopersicum	CBL1-1	SL1.00sc07408-86.1.1	Sol Genomics Network	[illegible]
Solanum	lycopersicum	CBL1-2	SL1.00sc05084-49.1.1	Sol Genomics Network	[illegible]
Solanum	lycopersicum	CBL10	SL1.00sc00576-14.1.1	Sol Genomics Network	[illegible]
Solanum	tuberosum	Unchar	Q38JG9	UniProt	[illegible]
Sorghum	bicolor	CBL01	C4P7Y0	UniProt	[illegible]
Sorghum	bicolor	CBL02	C4P7Y5	UniProt	[illegible]
Sorghum	bicolor	CBL03	C4P7Y6	UniProt	[illegible]
Sorghum	bicolor	CBL04	C4P7Y2	UniProt	[illegible]
Sorghum	bicolor	CBL05	C4P7Y4	UniProt	[illegible]
Sorghum	bicolor	CBL06	ACQ83550	NCBI GenBank	[illegible]
Sorghum	bicolor	CBL07	ACQ83548	NCBI GenBank	[illegible]
Sorghum	bicolor	CBL08	ACQ83554	NCBI GenBank	[illegible]
Thellungiella	halophila	CAsensor	A8IMA1	UniProt	[illegible]
Vicia	faba	CBL00	B2ZYT8	UniProt	[illegible]
Vitis	vinifera	CBL01	C4P7Y8	UniProt	[illegible]
Vitis	vinifera	CBL02	A5BYS8	UniProt	[illegible]
Vitis	vinifera	CBL03	A5AHA7	UniProt	[illegible]
Vitis	vinifera	CBL04	A5C1G1	UniProt	[illegible]
Vitis	vinifera	CBL05	C4P7Z2	UniProt	[illegible]
Vitis	vinifera	CBL06	ACQ83560	NCBI GenBank	[illegible]
Vitis	vinifera	CBL07	C4P7Z4	UniProt	[illegible]
Vitis	vinifera	CBL08	C4P7Z5	UniProt	[illegible]
Welwitschia	mirabilis	CBL01	gb|CV002984.1|	NCBI GenBank	[illegible]
Zamia	vasquezii	CBL01	gb|FD775526.1|	NCBI GenBank	[illegible]
Zea	mays	CBL00	ACG45209	NCBI GenBank	[illegible]
Zea	mays	CBL00	ACJ65322	NCBI GenBank	[illegible]
Zea	mays	CBL00	B6T8N9	UniProt	[illegible]
Zea	mays	CBL00	B6TXZ0	UniProt	[illegible]
Zea	mays	CBL00	C3TRS1	UniProt	[illegible]
Zea	mays	CBL00	C3TRS3	UniProt	[illegible]
Zea	mays	CBL03	B6TBI3	UniProt	[illegible]
Zea	mays	CBL04	B6TKT3	UniProt	[illegible]
Zea	mays	CBL09	B6T6B2	UniProt	[illegible]
Zingiber	officinale	CBL01	gi|87076007	NCBI GenBank	[illegible]

Species	Genus	Description	ID / Accession	Source	Sequence (residues included in MSA)
Adiantum	capillus-veneris	CIPK01	BP914063.1|DK958821.1|	NCBI GenBank	[illegible]
Adiantum	capillus-veneris	CIPK02	DK952883.1|DK944679.1|	NCBI GenBank	[illegible]
Adiantum	capillus-veneris	CIPK04	DK962775.1|	NCBI GenBank	[illegible]
Adiantum	capillus-veneris	CIPK03	DK962723.1|	NCBI GenBank	[illegible]
Amborella	trichopoda	CIPK04	FD440437.1|CD482986.1|	NCBI GenBank	[illegible]
Amborella	trichopoda	CIPK03	CO997150.1|CO999011.1|	NCBI GenBank	[illegible]
Amborella	trichopoda	CIPK02	CV006808.1|CD482728.1|	NCBI GenBank	[illegible]
Amborella	trichopoda	CIPK01	FD434646.1|CV006721.1|FD441700.1|	NCBI GenBank	[illegible]
Amborella	trichopoda	CIPK05	FD426386.1|	NCBI GenBank	[illegible]
Amborella	trichopoda	CIPK06	FD436643.1|	NCBI GenBank	[illegible]
Arabidopsis	thaliana	CIPK10	Q9C562	UniProt	[illegible]
Arabidopsis	thaliana	CIPK02	Q9LYQ8	UniProt	[illegible]
Arabidopsis	thaliana	CIPK20	Q9FJ54	UniProt	[illegible]
Arabidopsis	thaliana	CIPK12	Q9SN43	UniProt	[illegible]
Arabidopsis	thaliana	CIPK19	Q9FJ55	UniProt	[illegible]
Arabidopsis	thaliana	CIPK18	Q9LP51	UniProt	[illegible]
Arabidopsis	thaliana	CIPK13	O22971	UniProt	[illegible]
Arabidopsis	thaliana	CIPK06	O65554	UniProt	[illegible]
Arabidopsis	thaliana	CIPK15	P92937	UniProt	[illegible]
Arabidopsis	thaliana	CIPK17	Q94C40	UniProt	[illegible]
Arabidopsis	thaliana	CIPK01	Q8RWC9	UniProt	[illegible]
Arabidopsis	thaliana	CIPK23	Q93VD3	UniProt	[illegible]
Arabidopsis	thaliana	CIPK03	Q2V452	UniProt	[illegible]
Arabidopsis	thaliana	CIPK26	Q84VQ3	UniProt	[illegible]
Arabidopsis	thaliana	CIPK09	Q9MAM1	UniProt	[illegible]
Arabidopsis	thaliana	CIPK24	Q9LDI3	UniProt	[illegible]
Arabidopsis	thaliana	CIPK08	Q9STV4	[illegible]	[illegible]
Arabidopsis	thaliana	CIPK16	Q9SEZ7	[illegible]	[illegible]
Arabidopsis	thaliana	CIPK25	Q8W1D5	[illegible]	[illegible]
Arabidopsis	thaliana	CIPK05	Q9LEU7	[illegible]	[illegible]
Arabidopsis	thaliana	CIPK11	O22932	[illegible]	[illegible]
Arabidopsis	thaliana	CIPK14	Q9LZW4	[illegible]	[illegible]
Arabidopsis	thaliana	CIPK22	O80902	[illegible]	[illegible]
Arabidopsis	thaliana	CIPK04	Q9SUL7	[illegible]	[illegible]
Arabidopsis	thaliana	CIPK07	Q9XIW0	[illegible]	[illegible]
Arabidopsis	thaliana	CIPK21	Q94CG0	[illegible]	[illegible]
Bathycoccus	prasinos	CIPK01	K8EBQ8	[illegible]	[illegible]
Brachypodium	distachyon	CIPK04	Bradi1g17820.1	[illegible]	[illegible]
Brachypodium	distachyon	CIPK08	Bradi1g63080.1	[illegible]	[illegible]
Brachypodium	distachyon	CIPK14	Bradi2g06550.1	[illegible]	[illegible]
Brachypodium	distachyon	CIPK19	Bradi2g32590.1	[illegible]	[illegible]
Brachypodium	distachyon	CIPK23	Bradi2g53670.1	[illegible]	[illegible]
Brachypodium	distachyon	CIPK24	Bradi3g04630.1	[illegible]	[illegible]
Brachypodium	distachyon	CIPK18	Bradi2g22350.1	[illegible]	[illegible]
Brachypodium	distachyon	CIPK17	Bradi2g19970.1	[illegible]	[illegible]
Brachypodium	distachyon	CIPK22	Bradi2g50440.1	[illegible]	[illegible]
Brachypodium	distachyon	CIPK27	Bradi3g36910.1	[illegible]	[illegible]
Brachypodium	distachyon	CIPK31	Bradi4g30390.1	[illegible]	[illegible]
Brachypodium	distachyon	CIPK28	Bradi4g25880.1	[illegible]	[illegible]
Brachypodium	distachyon	CIPK32	Bradi4g44460.1	[illegible]	[illegible]
Brachypodium	distachyon	CIPK15	Bradi2g11220.1	[illegible]	[illegible]
Brachypodium	distachyon	CIPK07	Bradi1g58400.1	[illegible]	[illegible]
Brachypodium	distachyon	CIPK29	Bradi4g26410.1	[illegible]	[illegible]
Brachypodium	distachyon	CIPK09	Bradi1g64010.1	[illegible]	[illegible]
Brachypodium	distachyon	CIPK02	Bradi1g17250.1	[illegible]	[illegible]
Brachypodium	distachyon	CIPK11	Bradi1g76760.1	[illegible]	[illegible]
Brachypodium	distachyon	CIPK05	Bradi1g36520.1	[illegible]	[illegible]
Brachypodium	distachyon	CIPK20	Bradi2g40500.1	[illegible]	[illegible]
Brachypodium	distachyon	CIPK30	Bradi4g30380.1	[illegible]	[illegible]
Brachypodium	distachyon	CIPK13	Bradi2g06530.1	[illegible]	[illegible]
Brachypodium	distachyon	CIPK21	Bradi2g50430.1	[illegible]	[illegible]
Brachypodium	distachyon	CIPK06	Bradi1g38340.1	[illegible]	[illegible]
Brachypodium	distachyon	CIPK01	Bradi1g14200.1	[illegible]	[illegible]
Brachypodium	distachyon	CIPK26	Bradi3g05790.2	[illegible]	[illegible]
Brachypodium	distachyon	CIPK03	Bradi1g17810.1	[illegible]	[illegible]
Brassica	napus	CIPK00	Q8W2D7	[illegible]	[illegible]
Brassica	juncea	CIPK-like	A2IBU0	[illegible]	[illegible]
Ceratopteris	richardii	CIPK01	CV734872.1|	[illegible]	[illegible]
Chaetosphaeridium	globosum	CIPK01	PRJNA51161	[illegible]	[illegible]
Chamaecyparis	obtusa	CIPK02	BW992194.1|	[illegible]	[illegible]
Chamaecyparis	obtusa	CIPK01	BW988780.1|BW988823.1|BW991527.1|	[illegible]	[illegible]
Chlorella	sp	CIPK01	C4P7Q5	[illegible]	[illegible]
Cicer	arietinum	CIPK06	C0IRD0	[illegible]	[illegible]
Coleochaete	orbicularis	CIPK01	PRJNA51149	[illegible]	[illegible]
Cryptomeria	japonica	CIPK08	BJ937490.1|	[illegible]	[illegible]
Cryptomeria	japonica	CIPK05	BY878139.1|	[illegible]	[illegible]
Cryptomeria	japonica	CIPK01	BY890971.1|BY885416.1|	[illegible]	[illegible]
Cryptomeria	japonica	CIPK04	BW993447.1|BW993446.1|	[illegible]	[illegible]
Cryptomeria	japonica	CIPK03	BY895373.1|BY894718.1|	[illegible]	[illegible]
Cryptomeria	japonica	CIPK06	BY879961.1|	[illegible]	[illegible]
Cryptomeria	japonica	CIPK02	BY893993.1|BY891353.1|	[illegible]	[illegible]
Cryptomeria	japonica	CIPK07	BY890649.1|	[illegible]	[illegible]
Cycas	rumphii	CIPK01	EX920284.1|EX919856.1|DR062671.1|	[illegible]	[illegible]
Cycas	rumphii	CIPK03	DR062924.1|EX927250.1|	[illegible]	[illegible]
Cycas	rumphii	CIPK02	EX926704.1|EX917706.1|CB089566.1|	[illegible]	[illegible]
Cycas	rumphii	CIPK04	EX918702.1|	[illegible]	[illegible]
Ginkgo	biloba	CIPK01	EX936759.1|	[illegible]	[illegible]
Glycine	max	CIPK05	Gm0021x00079 (I1N370)	[illegible]	[illegible]
Glycine	max	CIPK12	Gm0015x00295 (I1JHK8)	[illegible]	[illegible]
Glycine	max	CIPK18	Gm0136x00060 (I1MZS7)	[illegible]	[illegible]
Glycine	max	CIPK19	Gm0194x00038 (I1LLV4)	[illegible]	[illegible]
Glycine	max	CIPK11	Gm0121x00190 (XP_003552138.1)	[illegible]	[illegible]
Glycine	max	CIPK15	Gm0139x00028 (K7LRS9)	[illegible]	[illegible]
Glycine	max	CIPK04	Gm0070x00164 (K7M1A3)	[illegible]	[illegible]
Glycine	max	CIPK03	Gm0003x00216 (XP_003524205)	[illegible]	[illegible]
Glycine	max	CIPK01	Gm0015x00035 (XP_003519143)	[illegible]	[illegible]
Glycine	max	CIPK02	Gm0025x00644 (XP_003549549)	[illegible]	[illegible]
Glycine	max	CIPK-like	Q8LK24	[illegible]	[illegible]
Glycine	max	CIPK16	Gm0030x00322 (I1M1S7)	[illegible]	[illegible]
Glycine	max	Predicted	C6TIM8	[illegible]	[illegible]
Glycine	max	CIPK30	Gm0010x00489 (I1L1Y4)	[illegible]	[illegible]
Glycine	max	CIPK06	Gm0010x00601.1 (I1L269)	[illegible]	[illegible]
Glycine	max	Predicted	C6TD54	[illegible]	[illegible]
Glycine	max	CIPK25	Gm0090x00407 (K7M4U3)	[illegible]	[illegible]
Glycine	max	CIPK21	Gm0076x00045.2 (I1JS31)	[illegible]	[illegible]
Glycine	max	CIPK23	Gm0161x00064 (I1MKE2)	[illegible]	[illegible]
Glycine	max	CIPK24	Gm0149x00160 (I1KHQ9)	[illegible]	[illegible]
Glycine	max	CIPK22	Gm0126x00060 (I1LZX1)	[illegible]	[illegible]
Glycine	max	CIPK26	Gm0069x00114 (I1JV21)	[illegible]	[illegible]
Glycine	max	CIPK28	Gm0015x00165 (XP_003519209.1)	[illegible]	[illegible]
Glycine	max	CIPK17	Gm0026x00538 (I1K8M8)	[illegible]	[illegible]
Glycine	max	CIPK29	Gm0113x00108 (I1KVL7)	[illegible]	[illegible]
Glycine	max	CIPK31	Gm0107x00186 (I1KGT5)	[illegible]	[illegible]
Glycine	max	CIPK27	Gm0088x00399 (XP_003547057.1)	[illegible]	[illegible]
Glycine	max	CIPK32	Gm0255x00036 (K7M8Q6)	[illegible]	[illegible]
Glycine	max	CIPK08	Gm0156x00061 (XP_003544927)	[illegible]	[illegible]
Glycine	max	CIPK10	Gm0015x00296 (I1JHK9)	[illegible]	[illegible]
Glycine	max	CIPK13	Gm0194x00043 (I1MZS1)	[illegible]	[illegible]
Glycine	max	CIPK34	Gm0075x00055 (I1L723)	[illegible]	[illegible]
Glycine	max	Kinase	C6F1T7	[illegible]	[illegible]
Glycine	max	CIPK36	Gm0085x00033 (I1NIA9)	[illegible]	[illegible]
Gnetum	gnemon	CIPK01	EX944558.1|	[illegible]	[illegible]
Gossypium	hirsutum	Predicted	Q6DXR4	[illegible]	[illegible]
Gossypium	hirsutum	CIPK00	A5YAI9	[illegible]	[illegible]
Hordeum	brevisubulatum	CIPK00	A5HLX6	[illegible]	[illegible]
Klebsormidium	flaccidum	CIPK01	PRJNA51159	[illegible]	[illegible]
Lepidium	latifolium	CIPK00	B7U1M7	[illegible]	[illegible]
Lotus	japonicus	Kinase	Q53VM3	[illegible]	[illegible]
Lotus	japonicus	Kinase	Q53UK3	[illegible]	[illegible]
Lotus	japonicus	Kinase	Q53VM2	[illegible]	[illegible]
Medicago	truncatula	Predicted	B7FM39	[illegible]	[illegible]
Mesembryanthemum	crystallinum	Predicted	Q9XFJ3	[illegible]	[illegible]
Mimulus	guttatus	CIPK12	mgf012372m	[illegible]	[illegible]
Mimulus	guttatus	CIPK08	mgf009289m	[illegible]	[illegible]
Mimulus	guttatus	CIPK05	mgf007182m	[illegible]	[illegible]
Mimulus	guttatus	CIPK09	mgf010314m	[illegible]	[illegible]
Mimulus	guttatus	CIPK18	mgf018800m	[illegible]	[illegible]
Mimulus	guttatus	CIPK11	mgf012289m	[illegible]	[illegible]
Mimulus	guttatus	CIPK06	mgf007928m	[illegible]	[illegible]
Mimulus	guttatus	CIPK10	mgf011351m	[illegible]	[illegible]
Mimulus	guttatus	CIPK03	mgf004012m	[illegible]	[illegible]
Mimulus	guttatus	CIPK04	mgf006200m	[illegible]	[illegible]
Mimulus	guttatus	CIPK15	mgf014590m	[illegible]	[illegible]
Mimulus	guttatus	CIPK16	mgf015424m	[illegible]	[illegible]
Mimulus	guttatus	CIPK13	mgf012602m	[illegible]	[illegible]
Mimulus	guttatus	CIPK20	mgf021508m	[illegible]	[illegible]
Mimulus	guttatus	CIPK19	mgf019687m	[illegible]	[illegible]
Mimulus	guttatus	CIPK17	mgf015484m	[illegible]	[illegible]
Nicotiana	tabacum	Kinase	Q2LAG3	[illegible]	[illegible]
Nicotiana	tabacum	Predicted	Q84QE0	[illegible]	[illegible]
Oryza	sativa	CIPK18	Q5W736	[illegible]	[illegible]
Oryza	sativa	CIPK02	Q7X996	[illegible]	[illegible]
Oryza	sativa	CIPK10	Q10LQ2	[illegible]	[illegible]
Oryza	sativa	CIPK05	Q9LWM4	[illegible]	[illegible]
Oryza	sativa	CIPK11	Q0JI49	[illegible]	[illegible]
Oryza	sativa	CIPK26	Q6H7U5	[illegible]	[illegible]
Oryza	sativa	CIPK28	A3B529	[illegible]	[illegible]
Oryza	sativa	CIPK12	Q5JLS2	[illegible]	[illegible]
Oryza	sativa	CIPK19	Q68Y49	[illegible]	[illegible]
Oryza	sativa	CIPK20	Q60EY8	[illegible]	[illegible]
Oryza	sativa	CIPK06	Q6Z9F4	[illegible]	[illegible]
Oryza	sativa	CIPK27	Q6ERS0	[illegible]	[illegible]
Oryza	sativa	CIPK14	Q2QYM3	[illegible]	[illegible]
Oryza	sativa	CIPK15	Q2RBF0	[illegible]	[illegible]
Oryza	sativa	CIPK17	Q75L42	[illegible]	[illegible]
Oryza	sativa	CIPK01	Q9LGV5	[illegible]	[illegible]
Oryza	sativa	CIPK21	Q0D4B2	[illegible]	[illegible]
Oryza	sativa	CIPK23	Q6ZLP5	[illegible]	[illegible]
Oryza	sativa	CIPK32	Q2QY53	[illegible]	[illegible]
Oryza	sativa	CIPK33	Q2RAX3	[illegible]	[illegible]
Oryza	sativa	CIPK31	Q6X4A2	[illegible]	[illegible]
Oryza	sativa	CIPK03	Q8LIG4	[illegible]	[illegible]
Oryza	sativa	CIPK09	Q10SC8	[illegible]	[illegible]
Oryza	sativa	CIPK24	Q69Q47	[illegible]	[illegible]
Oryza	sativa	CIPK08	Q5JLD8	[illegible]	[illegible]
Oryza	sativa	CIPK16	Q6ERS4	[illegible]	[illegible]
Oryza	sativa	CIPK13	Q5QNM6	[illegible]	[illegible]
Oryza	sativa	CIPK30	Q5JLQ9	[illegible]	[illegible]
Oryza	sativa	CIPK25	Q5Z6X0	[illegible]	[illegible]
Oryza	sativa	CIPK04	Q2QMI0	[illegible]	[illegible]
Oryza	sativa	CIPK07	Q75GK4	[illegible]	[illegible]
Oryza	sativa	Predicted	Q6ETM9	[illegible]	[illegible]
Oryza	sativa	CIPK22	Q5KQF5	[illegible]	[illegible]
Oryza	sativa	CIPK29	Q7XIW5	[illegible]	[illegible]
Ostreococcus	tauri	CIPK01	Q00XA0	[illegible]	[illegible]
Ostreococcus	lucimarinus	CIPK01	A4S6B2	[illegible]	[illegible]
Penium	margaritaceum	CIPK01	PRJNA68813	[illegible]	[illegible]
Persea	americana	Kinase	B0FM60	[illegible]	[illegible]
Phaseolus	vulgaris	Kinase	B0I554	[illegible]	[illegible]
Phaseolus	vulgaris	Kinase	B0I557	[illegible]	[illegible]
Phaseolus	vulgaris	Kinase	B0I556	[illegible]	[illegible]
Phaseolus	vulgaris	Kinase	B0I555	[illegible]	[illegible]
Phaseolus	vulgaris	Kinase	B0I553	[illegible]	[illegible]
Physcomitrella	patens	CIPK01	TK1 (KF310898)	[illegible]	[illegible]
Physcomitrella	patens	CIPK05	C4P7R0	[illegible]	[illegible]
Physcomitrella	patens	CIPK02	C4P7Q7	[illegible]	[illegible]
Physcomitrella	patens	CIPK03	C4P7Q8	[illegible]	[illegible]
Physcomitrella	patens	CIPK04	C4P7Q9	[illegible]	[illegible]
Physcomitrella	patens	CIPK06	TK6 (KF310899)	[illegible]	[illegible]
Physcomitrella	patens	CIPK07	TK7 (KF310900)	[illegible]	[illegible]
Picea	sitchensis	Predicted	A9NWA3	[illegible]	[illegible]
Picea	sitchensis	CIPK03	GW724668.1|ES874922.1|	[illegible]	[illegible]
Picea	glauca	CIPK04	GE481941.1|DV987117.2|EX420957.1|EX307400.1|EX332250.1|	[illegible]	[illegible]
Picea	sitchensis	CIPK04	DR496520.1|DR502389.1|	[illegible]	[illegible]
Picea	glauca	CIPK02	GT052021.1|EX395604.1|DR553933.1|GR953982.1|DV971838.2|	[illegible]	[illegible]
Picea	sitchensis	CIPK06	DR510063.1|ES866924.1|	[illegible]	[illegible]
Picea	glauca	CIPK03	EX377784.1|DV975038.2|DV981975.2|DV981459.2|GO362998.1|	[illegible]	[illegible]
Picea	glauca	CIPK14	EX427307.1|	[illegible]	[illegible]
Picea	sitchensis	Predicted	A9NWN4	[illegible]	[illegible]
Picea	glauca	CIPK06	EX406376.1|EX405339.1|EX422233.1|EX377949.1|	[illegible]	[illegible]
Picea	sitchensis	CIPK11	DR523201.1|	[illegible]	[illegible]
Picea	glauca	CIPK12	EX395632.1|GO364249.1|	[illegible]	[illegible]
Picea	sitchensis	CIPK07	DR488323.1|	[illegible]	[illegible]
Picea	sitchensis	Predicted	B8LQ83	[illegible]	[illegible]
Picea	glauca	CIPK01	EX429066.1|EX321787.1|EX336363.1|EX424514.1|EX384215.1|GO368866.1|	[illegible]	[illegible]
Picea	sitchensis	CIPK01	ES260282.1|GW716040.1|ES854133.1|ES878367.1|DR485594.1|DR525136.1|DR507086.1|DR492560.1|	[illegible]	[illegible]
Picea	glauca	CIPK09	DV971723.2|GT739300.1|GO364658.1|	[illegible]	[illegible]
Picea	glauca	CIPK08	EX308593.1|EX377822.1|EX358179.1|	[illegible]	[illegible]
Picea	glauca	CIPK15	EX355497.1|	[illegible]	[illegible]
Picea	sitchensis	CIPK02	ES259329.1|ES248891.1|ES259285.1|	[illegible]	[illegible]
Picea	glauca	CIPK07	DV995522.2|EX443696.1|GO371693.1|EX368020.1|	[illegible]	[illegible]
Picea	sitchensis	Predicted	A9NWP7	[illegible]	[illegible]
Picea	sitchensis	CIPK10	DR533050.1|	[illegible]	[illegible]
Picea	glauca	CIPK05	EX355761.1|EX351514.1|EX379301.1|EX378637.1|EX379005.1|	[illegible]	[illegible]
Picea	glauca	CIPK11	EX357503.1|GO361056.1|CK438183.2|	[illegible]	[illegible]
Picea	sitchensis	CIPK05	DR525097.1|ES860451.1|	[illegible]	[illegible]
Picea	sitchensis	CIPK08	DR507050.1|	[illegible]	[illegible]
Picea	sitchensis	CIPK09	GH288155.1|	[illegible]	[illegible]
Picea	glauca	CIPK13	EX374353.1|EX374718.1|	[illegible]	[illegible]
Picea	glauca	CIPK10	GE477126.1|DV995416.1|DV978670.2|	[illegible]	[illegible]
Pinus	taeda	CIPK02	DR182380.1|CF386583.1|BE049769.1|DR165100.1|DR178546.1|DR180840.1|DR011875.1|CF396859.1|BF220442.1|CF668138.1|[illegible]	[illegible]	[illegible]
Pinus	taeda	CIPK05	CF666888.1|DR161128.1|CF387106.1|DR102516.1|CF388001.1|CO197374.1|DR745216.1|CO167071.1|CF393993.1|DR112206.1|[illegible]	[illegible]	[illegible]
Pinus	contorta	CIPK02	GT245648.1|	[illegible]	[illegible]
Pinus	pinaster	CIPK01	CR393032.1|BX677295.1|CR393394.1|BX252872.1|	[illegible]	[illegible]
Pinus	contorta	CIPK01	GT230005.1|	[illegible]	[illegible]
Pinus	taeda	CIPK01	DN461068.1|DN458660.1|CO363418.1|CV139147.1|DR385591.1|DN460665.1|CN784748.1|DN462529.1|DN458909.1|DN457155.1[illegible]	[illegible]	[illegible]
Pinus	pinaster	CIPK02	CT576730.1|	[illegible]	[illegible]
Pinus	taeda	CIPK10	CN783836.1|DT625802.1|CO368613.1|DT624699.1|DN450083.1|	[illegible]	[illegible]
Pinus	taeda	CIPK14	DR388492.1|	[illegible]	[illegible]
Pinus	taeda	CIPK11	DR110082.1|BM158660.1|BF777807.1|	[illegible]	[illegible]
Pinus	taeda	CIPK06	CO200055.1|CO200140.1|	[illegible]	[illegible]
Pinus	taeda	CIPK15	CO200166.1|	[illegible]	[illegible]
Pinus	contorta	CIPK04	GT255135.1|	[illegible]	[illegible]
Pinus	pinaster	CIPK03	GW575388.1|	[illegible]	[illegible]
Pinus	taeda	CIPK04	DR058223.1|CF472415.1|DR081667.1|CO170267.1|DR060931.1|DR057892.1|	[illegible]	[illegible]
Pinus	taeda	CIPK09	DR685470.1|DR689197.1|DT635149.1|	[illegible]	[illegible]
Pinus	taeda	CIPK07	DT636525.1|DT639050.1|DR695139.1|	[illegible]	[illegible]
Pinus	taeda	CIPK08	AW011235.1|DT637200.1|DR692470.1|	[illegible]	[illegible]
Pinus	contorta	CIPK03	GT253099.1|	[illegible]	[illegible]
Pinus	taeda	CIPK13	DR058233.1|	[illegible]	[illegible]
Pinus	taeda	CIPK12	DR098231.1|	[illegible]	[illegible]
Pinus	taeda	CIPK16	BM428492.1|	[illegible]	[illegible]
Pinus	taeda	CIPK03	CD028119.1|AW289582.1|	[illegible]	[illegible]
Pinus	pinaster	CIPK04	CT576379.1|	[illegible]	[illegible]
Pisum	sativum	CIPK00	Q84XC0	[illegible]	[illegible]
Populus	trichocarpa	CIPK22	A0MNK8	[illegible]	[illegible]
Populus	trichocarpa	Predicted	B9IPI5	[illegible]	[illegible]
Populus	trichocarpa	CIPK02	A0MNJ0	[illegible]	[illegible]
Populus	trichocarpa	CIPK14	A0MNK1	[illegible]	[illegible]
Populus	trichocarpa	CIPK11	A0MNJ8	[illegible]	[illegible]
Populus	trichocarpa	CIPK17	A0MNK4	[illegible]	[illegible]
Populus	trichocarpa	CIPK18	A0MNK5	[illegible]	[illegible]
Populus	trichocarpa	CIPK19	A0MNK6	[illegible]	[illegible]
Populus	trichocarpa	CIPK07	A0MNJ4	[illegible]	[illegible]
Populus	trichocarpa	CIPK09	A0MNJ6	[illegible]	[illegible]
Populus	trichocarpa	CIPK01	A0MNI9	[illegible]	[illegible]
Populus	trichocarpa	CIPK24	A0MNL0	[illegible]	[illegible]
Populus	trichocarpa	CIPK25	A0MNL1	[illegible]	[illegible]
Populus	trichocarpa	CIPK03	A0MNJ1	[illegible]	[illegible]
Populus	trichocarpa	CIPK04	A0MNJ2	[illegible]	[illegible]
Populus	trichocarpa	CIPK12	A0MNJ9	[illegible]	[illegible]
Populus	trichocarpa	CIPK13	A0MNK0	[illegible]	[illegible]
Populus	trichocarpa	CIPK26	A0MNL2	[illegible]	[illegible]
Populus	trichocarpa	CIPK27	A0MNL3	[illegible]	[illegible]
Populus	trichocarpa	CIPK06	A0MNL4	[illegible]	[illegible]
Populus	trichocarpa	CIPK20	A0MNL5	[illegible]	[illegible]
Populus	trichocarpa	CIPK23	A0MNK9	[illegible]	[illegible]
Populus	trichocarpa	CIPK15	A0MNK2	[illegible]	[illegible]
Populus	trichocarpa	CIPK16	A0MNK3	[illegible]	[illegible]
Populus	trichocarpa	CIPK21	A0MNK7	[illegible]	[illegible]
Populus	trichocarpa	CIPK05	A0MNJ3	[illegible]	[illegible]
Populus	trichocarpa	CIPK10	A0MNJ7	[illegible]	[illegible]
Populus	trichocarpa	Predicted	B9IM29	[illegible]	[illegible]
Prunus	persica	CIPK03	ppa004815m	[illegible]	[illegible]
Prunus	persica	CIPK07	ppa005370m	[illegible]	[illegible]
Prunus	persica	CIPK08	ppa005839m	[illegible]	[illegible]
Prunus	persica	CIPK02	ppa005365m	[illegible]	[illegible]
Prunus	persica	CIPK09	ppa004907m	[illegible]	[illegible]
Prunus	persica	CIPK01	ppa006023m	[illegible]	[illegible]
Prunus	persica	CIPK16	ppa005480m	[illegible]	[illegible]
Prunus	persica	CIPK04	ppa005492m	[illegible]	[illegible]
Prunus	persica	CIPK10	ppa005881m	[illegible]	[illegible]
Prunus	persica	CIPK12	ppa005700m	[illegible]	[illegible]
Prunus	persica	CIPK13	ppa005741m	[illegible]	[illegible]
Prunus	persica	CIPK15	ppa005163m	[illegible]	[illegible]
Prunus	persica	CIPK05	ppa005575m	[illegible]	[illegible]
Prunus	persica	CIPK14	ppa020118m	[illegible]	[illegible]
Prunus	persica	CIPK06	ppa005344m	[illegible]	[illegible]
Prunus	persica	CIPK11	ppa005902m	[illegible]	[illegible]
Prunus	persica	CIPK18	ppa006253m	[illegible]	[illegible]
Prunus	persica	CIPK17	ppa005267m	[illegible]	[illegible]
Pseudotsuga	menziesii	CIPK01	CN636134.1|	[illegible]	[illegible]
Pseudotsuga	menziesii	CIPK03	CN636205.1|	[illegible]	[illegible]
Pseudotsuga	menziesii	CIPK02	CN637423.1|	[illegible]	[illegible]
Ricinus	communis	CIPK-like	B9T650	[illegible]	[illegible]
Ricinus	communis	CIPK-like	B9S4C5	[illegible]	[illegible]
Ricinus	communis	CIPK-like	B9RVE7	[illegible]	[illegible]
Ricinus	communis	CIPK-like	B9S8A4	[illegible]	[illegible]
Ricinus	communis	CIPK-like	B9S8A5	[illegible]	[illegible]
Ricinus	communis	CIPK-like	B9T6V8	[illegible]	[illegible]
Ricinus	communis	CIPK-like	B9S009	[illegible]	[illegible]
Ricinus	communis	CIPK-like	B9T407	[illegible]	[illegible]
Ricinus	communis	CIPK-like	B9T2W2	[illegible]	[illegible]
Ricinus	communis	CIPK-like	B9SET9	[illegible]	[illegible]
Ricinus	communis	CIPK-like	B9SMX5	[illegible]	[illegible]
Ricinus	communis	CIPK-like	B9RNW5	[illegible]	[illegible]
Ricinus	communis	CIPK-like	B9SMT0	[illegible]	[illegible]
Ricinus	communis	CIPK-like	B9RVE0	[illegible]	[illegible]
Ricinus	communis	CIPK-like	B9T8H6	[illegible]	[illegible]
Ricinus	communis	CIPK-like	B9RTG4	[illegible]	[illegible]
Ricinus	communis	CIPK-like	B9T2E1	[illegible]	[illegible]
Selaginella	moellendorffii	CIPK03	C4P7R5	[illegible]	[illegible]
Selaginella	moellendorffii	CIPK02	C4P7R4	[illegible]	[illegible]
Selaginella	moellendorffii	CIPK01	C4P7R3	[illegible]	[illegible]
Selaginella	moellendorffii	CIPK04	C4P7R6	[illegible]	[illegible]
Selaginella	moellendorffii	CIPK05	C4P7R7	[illegible]	[illegible]
Solanum	lycopersicum	CIPK01	SL1.00sc05805-154.1.1	[illegible]	[illegible]
Solanum	lycopersicum	CIPK03	SL1.00sc00226-236.1.1	[illegible]	[illegible]
Solanum	lycopersicum	CIPK11	SL1.00sc04337-310.1.1	[illegible]	[illegible]
Solanum	lycopersicum	CIPK02	SL1.00sc01651-6.1.1	[illegible]	[illegible]
Solanum	lycopersicum	CIPK04	SL1.00sc02227-337.1.1	[illegible]	[illegible]
Solanum	lycopersicum	CIPK06	SL1.00sc05103-118.1.1	[illegible]	[illegible]
Solanum	lycopersicum	CIPK14	SL1.00sc03897-110.1.1	[illegible]	[illegible]
Solanum	lycopersicum	CIPK00	Q4W3B3	[illegible]	[illegible]
Solanum	lycopersicum	CIPK10	SL1.00sc06303-71.1.1	[illegible]	[illegible]
Solanum	lycopersicum	CIPK05	SL1.00sc04548-81.1.1	[illegible]	[illegible]
Solanum	lycopersicum	CIPK07	SL1.00sc01435-132.1.1	[illegible]	[illegible]
Solanum	lycopersicum	CIPK09	SL1.00sc05805-153.1.1	[illegible]	[illegible]
Solanum	lycopersicum	CIPK12	SL1.00sc06183-390.1.1	[illegible]	[illegible]
Solanum	lycopersicum	CIPK13-1	SL1.00sc03668-105.1.1	[illegible]	[illegible]
Solanum	lycopersicum	CIPK13-2	SL1.00sc03668-104.1.1	[illegible]	[illegible]
Sorghum	bicolor	CIPK15	C4P7U2	[illegible]	[illegible]
Sorghum	bicolor	CIPK25	C4P7S7	[illegible]	[illegible]
Sorghum	bicolor	CIPK19	C4P7R9	[illegible]	[illegible]
Sorghum	bicolor	CIPK20	C4P7S9	[illegible]	[illegible]
Sorghum	bicolor	CIPK29	C4P7U9	[illegible]	[illegible]
Sorghum	bicolor	CIPK31	C4P7T4	[illegible]	[illegible]
Sorghum	bicolor	CIPK10 maybeFRAG	C4P7U4	[illegible]	[illegible]
Sorghum	bicolor	CIPK13	C4P7U5	[illegible]	[illegible]
Sorghum	bicolor	CIPK12	C4P7T3	[illegible]	[illegible]
Sorghum	bicolor	CIPK22	C4P7T7	[illegible]	[illegible]
Sorghum	bicolor	CIPK14	C4P7S4	[illegible]	[illegible]
Sorghum	bicolor	CIPK02	C4P7T9	[illegible]	[illegible]
Sorghum	bicolor	CIPK30	C4P7T5	[illegible]	[illegible]
Sorghum	bicolor	CIPK21	C4P7T1	[illegible]	[illegible]
Sorghum	bicolor	CIPK01	C4P7U1	[illegible]	[illegible]
Sorghum	bicolor	CIPK17	C4P7S5	[illegible]	[illegible]
Sorghum	bicolor	CIPK23	C4P7S2	[illegible]	[illegible]
Sorghum	bicolor	CIPK03	C4P7T6	[illegible]	[illegible]
Sorghum	bicolor	Kinase	O24343	[illegible]	[illegible]
Sorghum	bicolor	CIPK26	C4P7T8	[illegible]	[illegible]
Sorghum	bicolor	CIPK27	C4P7S0	[illegible]	[illegible]
Sorghum	bicolor	CIPK28	C4P7S8	[illegible]	[illegible]
Sorghum	bicolor	CIPK09	C4P7S1	[illegible]	[illegible]
Sorghum	bicolor	CIPK24	C4P7U7	[illegible]	[illegible]
Sorghum	bicolor	CIPK08	C4P7U8	[illegible]	[illegible]
Sorghum	bicolor	CIPK16	C4P7S3	[illegible]	[illegible]
Sorghum	bicolor	CIPK06	C4P7T2	[illegible]	[illegible]
Sorghum	bicolor	CIPK32	C4P7U6	[illegible]	[illegible]
Sorghum	bicolor	CIPK07	C4P7U0	[illegible]	[illegible]
Sorghum	bicolor	CIPK04	C4P7R8	[illegible]	[illegible]
Sorghum	bicolor	CIPK11	C4P7U3	[illegible]	[illegible]
Sorghum	bicolor	CIPK05	C4P7S6	[illegible]	[illegible]
Syntrichia	ruralis	CIPK02	CN205271.1|	[illegible]	[illegible]
Syntrichia	ruralis	CIPK01	CN206747.1|	[illegible]	[illegible]
Trifolium	pratense	Predicted	Q2PET0	[illegible]	[illegible]
Vicia	faba	CIPK01	B2ZYT7	[illegible]	[illegible]
Vigna	unguiculata	CIPK00	A7BJ79	[illegible]	[illegible]
Vitis	vinifera	CIPK07	A5BJJ8	[illegible]	[illegible]
Vitis	vinifera	CIPK13	C4P7W2	[illegible]	[illegible]
Vitis	vinifera	CIPK11	C4P7W0	[illegible]	[illegible]
Vitis	vinifera	CIPK16	C4P7W5	[illegible]	[illegible]
Vitis	vinifera	CIPK01	A5BKH1	[illegible]	[illegible]
Vitis	vinifera	CIPK03	A5C5K7	[illegible]	[illegible]
Vitis	vinifera	CIPK02 FRAG	C4P7V1	[illegible]	[illegible]
Vitis	vinifera	CIPK05	A5C5T4	[illegible]	[illegible]
Vitis	vinifera	CIPK19	C4P7W8	[illegible]	[illegible]
Vitis	vinifera	CIPK04	A5BQ82	[illegible]	[illegible]
Vitis	vinifera	CIPK18	C4P7W7	[illegible]	[illegible]
Vitis	vinifera	CIPK12	C4P7W1	[illegible]	[illegible]
Vitis	vinifera	CIPK09	A5BZV3	[illegible]	[illegible]
Vitis	vinifera	CIPK17	C4P7W6	[illegible]	[illegible]
Vitis	vinifera	CIPK15	C4P7W4	[illegible]	[illegible]
Vitis	vinifera	CIPK10	C4P7V9	[illegible]	[illegible]
Vitis	vinifera	CIPK08	A5BHI9	[illegible]	[illegible]
Vitis	vinifera	CIPK20	C4P7W9	[illegible]	[illegible]
Vitis	vinifera	CIPK14	C4P7W3	[illegible]	[illegible]
Vitis	vinifera	CIPK06	A5B4V8	[illegible]	[illegible]
Welwitschia	mirabilis	CIPK01	DT599903.1|DT597454.1|	[illegible]	[illegible]
Welwitschia	mirabilis	CIPK03	DT605058.1|	[illegible]	[illegible]
Welwitschia	mirabilis	CIPK02	DT587517.1|CV005649.1|	[illegible]	[illegible]
Zamia	vazquezii	CIPK02	FD768239.1|	[illegible]	[illegible]
Zamia	vazquezii	CIPK01	FD767951.1|	[illegible]	[illegible]
Zamia	vazquezii	CIPK03	FD768239.1|	[illegible]	[illegible]
Zea	mays	CIPK15	B6SW34	[illegible]	[illegible]
Zea	mays	CIPK15	B6SXL5	[illegible]	[illegible]
Zea	mays	CIPK15	B6SH63	[illegible]	[illegible]
Zea	mays	CIPK15	B6THP4	[illegible]	[illegible]
Zea	mays	CIPK15	B6SXI2	[illegible]	[illegible]
Zea	mays	Predicted	C0PK96	[illegible]	[illegible]
Zea	mays	CIPK11	B6SK56	[illegible]	[illegible]
Zea	mays	CIPK15	B6TX22	[illegible]	[illegible]
Zea	mays	Predicted	B4FA17	[illegible]	[illegible]
Zea	mays	CIPK15	B4FXZ1	[illegible]	[illegible]
Zea	mays	Predicted	C4IYR0	[illegible]	[illegible]
Zea	mays	CIPK01	B6TDE7	[illegible]	[illegible]
Zea	mays	CIPK01	B6T3T6	[illegible]	[illegible]
Zea	mays	CIPK01	B6SUD4	[illegible]	[illegible]
Zea	mays	Predicted	C0P7R9	[illegible]	[illegible]
Zea	mays	CIPK-like	B6SY58	[illegible]	[illegible]
Zea	mays	CIPK31	B5AU21	[illegible]	[illegible]
Zea	mays	Predicted	C0P6L2	[illegible]	[illegible]
Zea	mays	Predicted	B4FFI0	[illegible]	[illegible]
Zea	mays	Predicted	C0P610	[illegible]	[illegible]
Zea	mays	CIPK16	C3TRS5	[illegible]	[illegible]
Zea	mays	CIPK-like	B6TTX6	[illegible]	[illegible]
Zea	mays	Kinase	B6U5K8	[illegible]	[illegible]
Zea	mays	CIPK11	B6SPQ5	[illegible]	[illegible]

Gene	Forward Primer Name	Forward Primer 5' Gateway Extension (attB1)	Forward Primer Sequence	Reverse Primer Name	Reverse Primer 5' Gateway Extension (attB2)	Reverse Primer Sequence
PpCBL1	PpCBL1-F1g	GGGGACAAGTTTGTACAAAAAAGCAGGCTTC	ATGGGCTGTTTCCACTCC	PpCBL1-R2g	GGGGACCACTTTGTACAAGAAAGCTGGGTC	TTAGGCACTGCCATCATCC
PpCBL2	PpCBL2-F1g	GGGGACAAGTTTGTACAAAAAAGCAGGCTTC	ATGGGTTGCTATGGGTCCA	PpCBL2-R1g	GGGGACCACTTTGTACAAGAAAGCTGGGTC	TCAATTATCTTCGACCCGAGAG
PpCBL3	PpCBL3-F1g	GGGGACAAGTTTGTACAAAAAAGCAGGCTTC	ATGTGGAAGTTATTAGAGCTG	PpCBL3-R1g	GGGGACCACTTTGTACAAGAAAGCTGGGTC	TCAAGTTTCTTCTACTAAGG
PpCBL4	PpCBL4-F4g	GGGGACAAGTTTGTACAAAAAAGCAGGCTTC	ATGGGGGCTGCGGTG	PpCBL4-R1g	GGGGACCACTTTGTACAAGAAAGCTGGGTC	TCAATCTTTGTCAATTTTGCGAG
PpCIPK1	PpCIPK1-F1g	GGGGACAAGTTTGTACAAAAAAGCAGGCTTC	ATGAACGGCAACAACAATCGC	PpCIPK1-R1g	GGGGACCACTTTGTACAAGAAAGCTGGGTC	CAATAGTAAATCGCCTCCTTGGC
PpCIPK2	PpCIPK2-F1g	GGGGACAAGTTTGTACAAAAAAGCAGGCTTCTT	AGTGACCATGACTTCCACC	PpCIPK2-R1g	GGGGACCACTTTGTACAAGAAAGCTGGGTC	AATGCAATTCTAGTAAAGGTACAG
PpCIPK3	PpCIPK3-F1g	GGGGACAAGTTTGTACAAAAAAGCAGGCTTC	ATGACAACTAAATCAAACATGCC	PpCIPK3-R1g	GGGGACCACTTTGTACAAGAAAGCTGGGTC	TTTCTACTTTTGCTCACTTGTAGG
PpCIPK4	PpCIPK4-F1g	GGGGACAAGTTTGTACAAAAAAGCAGGCTTC	ATGACTACCGCGACACCAAG	PpCIPK4-R2g	GGGGACCACTTTGTACAAGAAAGCTGGGTC	CTACTTTTGTTCACATGCAGGAAGAG
PpCIPK5	PpCIPK5-F4g	GGGGACAAGTTTGTACAAAAAAGCAGGCTTC	ATGATGAATTCGAATAGTCTTCGC	PpCIPK5-R1g	GGGGACCACTTTGTACAAGAAAGCTGGGTC	TTACTTCAATGGGGAAACCTTG
PpCIPK6	PpCIPK6-F1g	GGGGACAAGTTTGTACAAAAAAGCAGGCTTC	ATGAGGAAAGTGGGCAAGTATGAAGTG	PpCIPK6-R1g	GGGGACCACTTTGTACAAGAAAGCTGGGTC	CAGCGGTATATCCTTGCTCCTCATC
PpCIPK7	PpCIPK7-F4g	GGGGACAAGTTTGTACAAAAAAGCAGGCTTC	GAGGAAGTGATGAGGAAGGTG	PpCIPK7-R1g	GGGGACCACTTTGTACAAGAAAGCTGGGTC	CCATACACACACACTTCTATGCTC
