# Supplementary material for: Comparative phylogenomics of the CBL-CIPK calcium-decoding network in the moss Physcomitrella, Arabidopsis, and other green lineages
Source: Front Plant Sci. 2014 May 14;5:187. doi: 10.3389/fpls.2014.00187 (PMC4030171; doi:10.3389/fpls.2014.00187)
Supplement: Supplementary file 3 [file Presentation2.PDF]

PpCIPK1-BD
CBL-AD

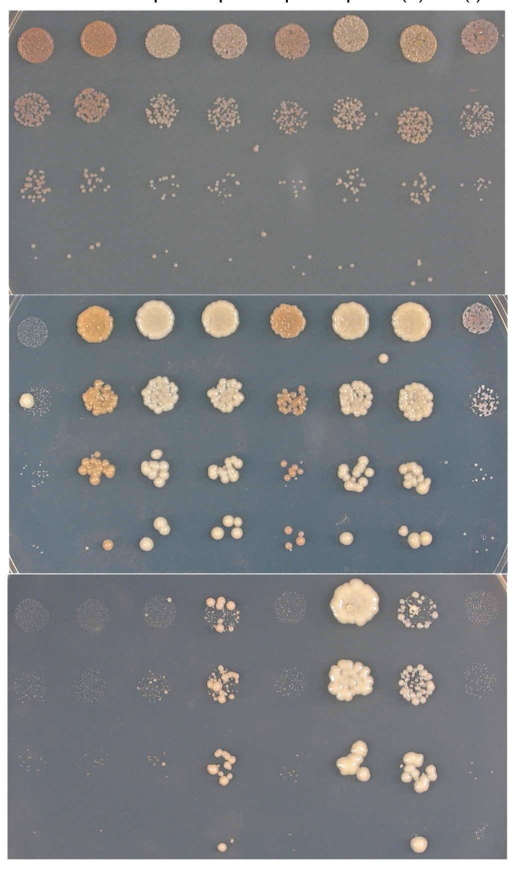


1

PpCIPK2-BD
CBL-AD

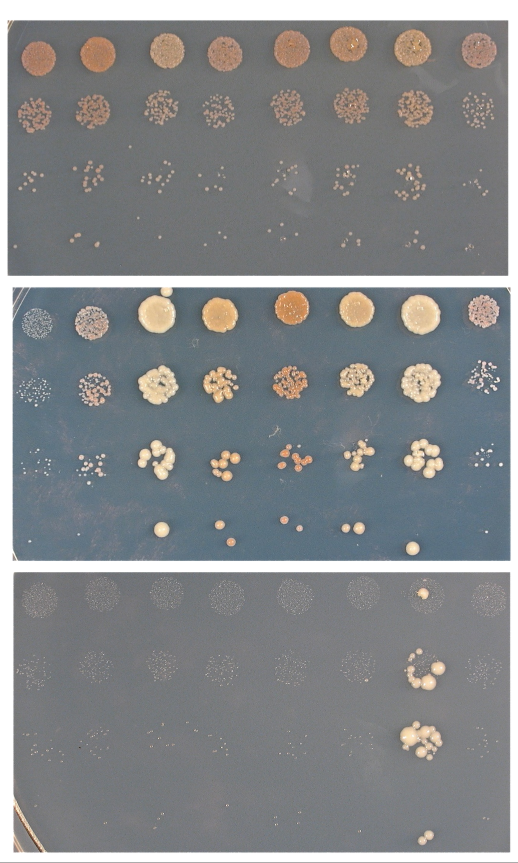


2

PpCIPK3-BD
CBL-AD

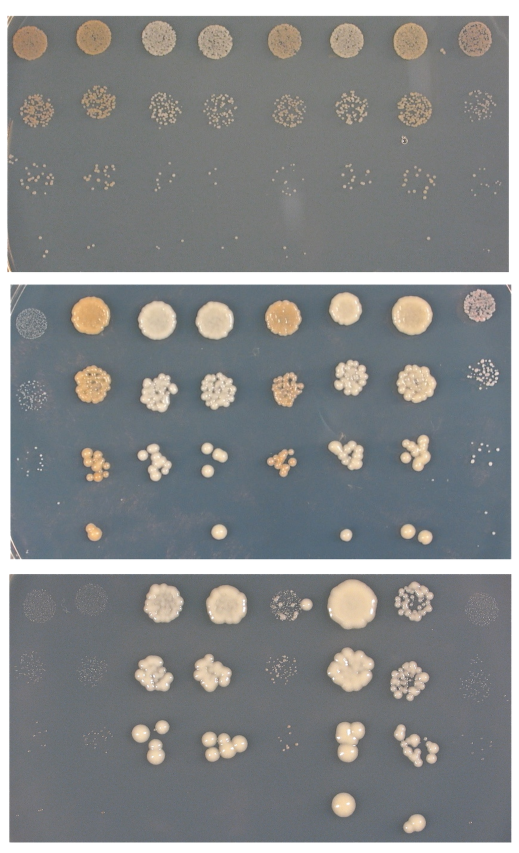


3

PpCIPK4-BD
CBL-AD

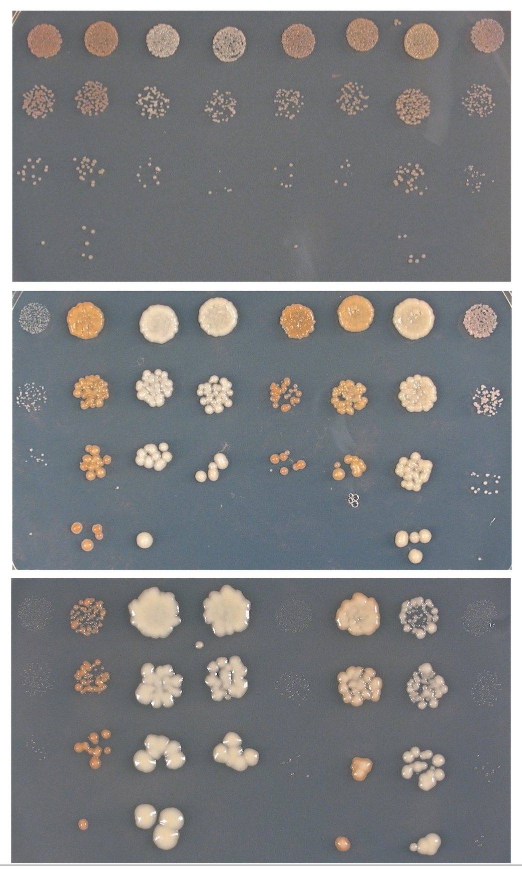


4

PpCIPK5-BD
CBL-AD

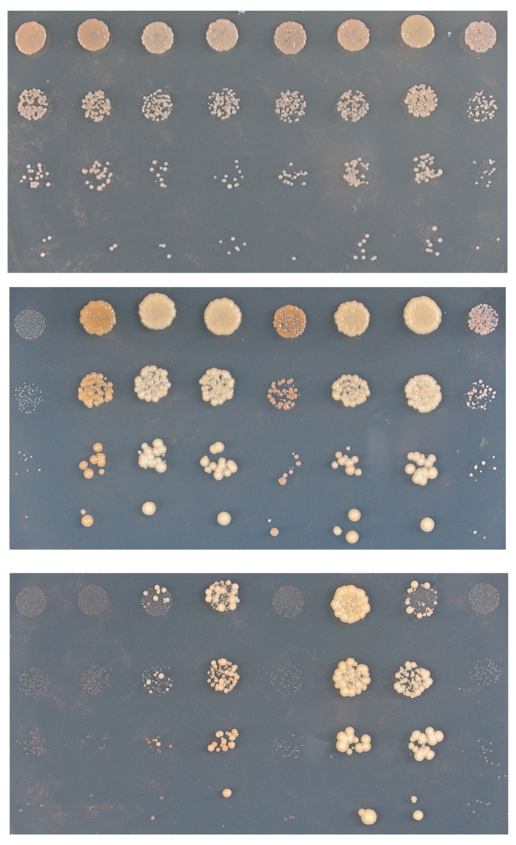


5

PpCIPK6-BD
CBL-AD

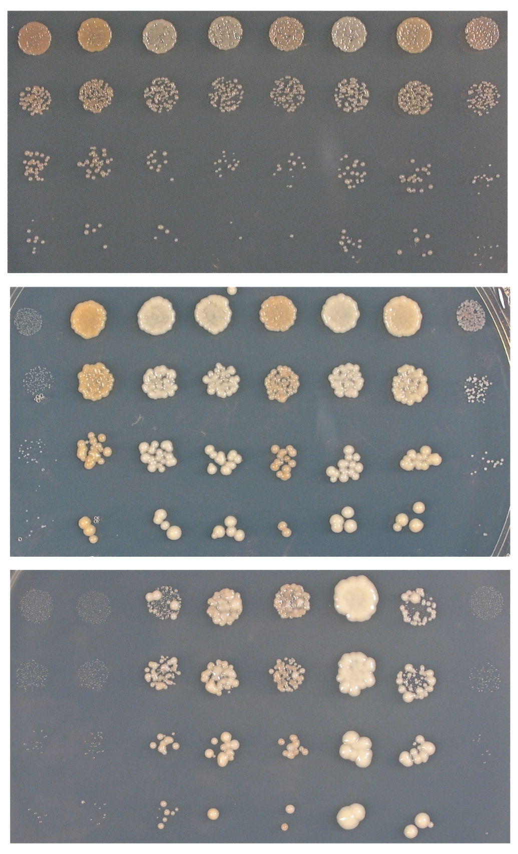


6

PpCIPK7-BD
CBL-AD

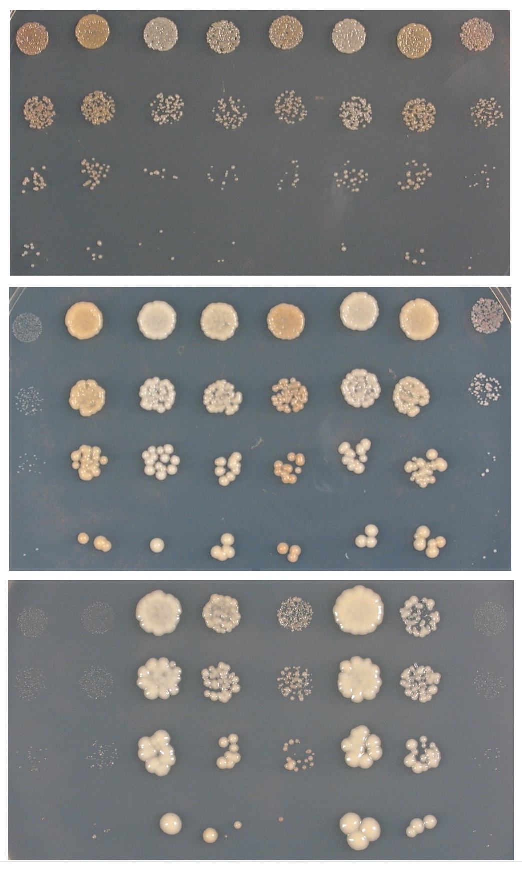


7

PpCBL1-BD
PpCIPK-AD

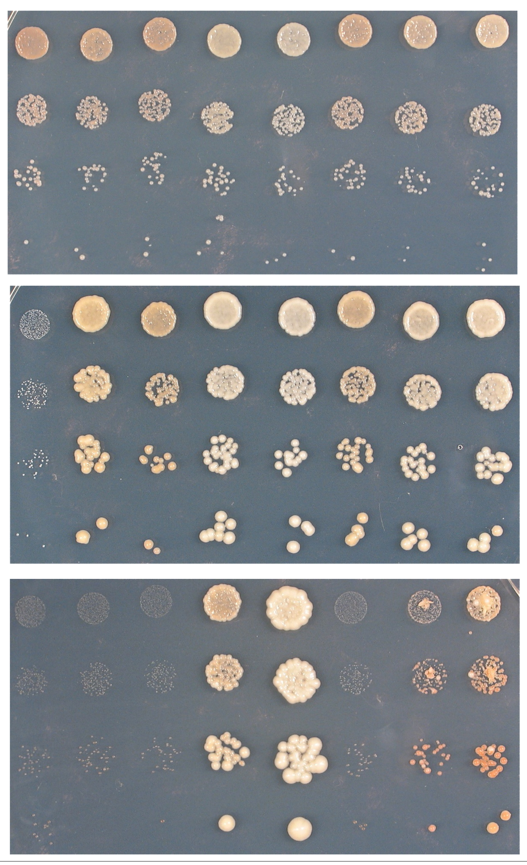


8

PpCBL2-BD
PpCIPK-AD

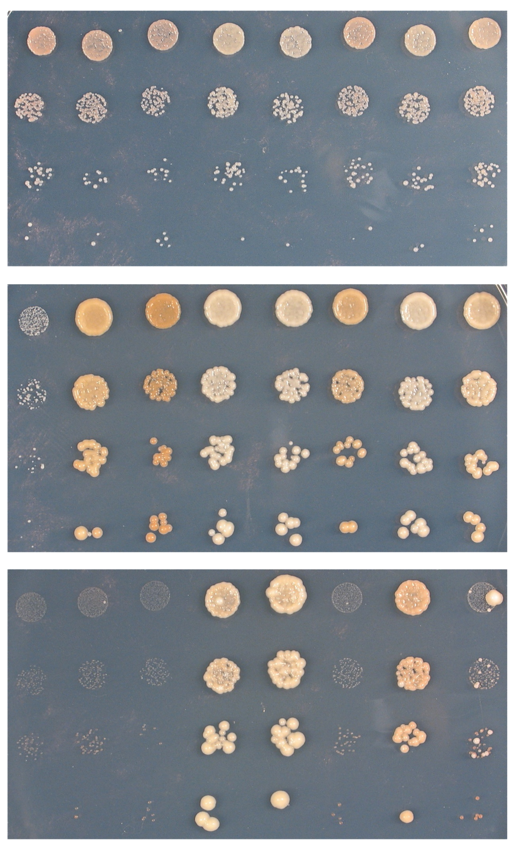


9

PpCBL3-BD
PpCIPK-AD

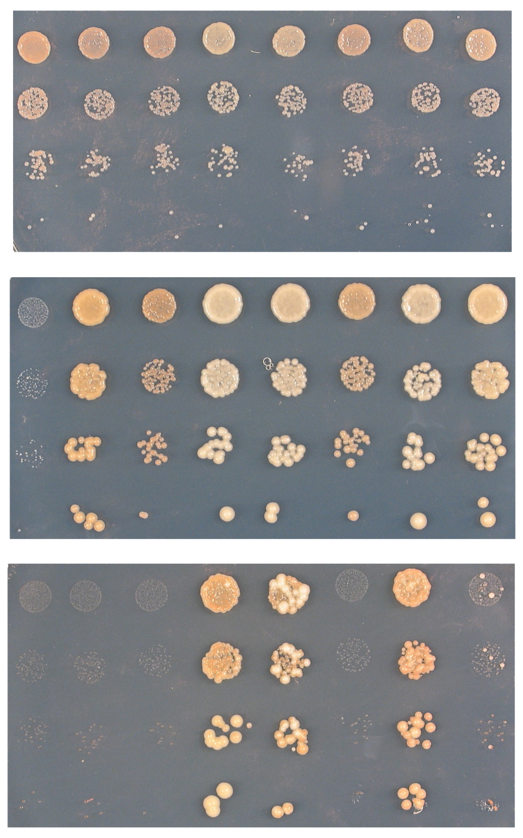


10

PpCBL4-BD
PpCIPK-AD

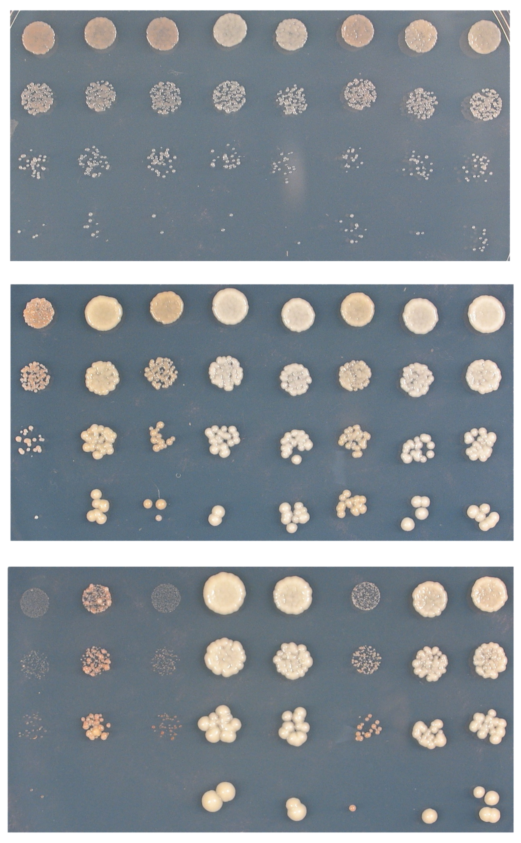


11

# Abbreviations

- **EV: empty vector**
- **At: *Arabidopsis thaliana***
- **Pp: *Physcomitrella patens***
  - **<u>Note</u>: numbers reference gene IDs in text and supp. tables S1 and S2**
- **[slides 1-7] (+): AtCBL4-AD + AtCIPK24-BD;**
- **[slides 1-7] (-): AtCBL4-AD + AtCIPK6-BD;**
- **$OD_{600}$: optical density at 600 nm (approximation of cell density)**
- **<u>Note</u>: images from different days are chosen for display to best illustrate differences in growth between different interacting pairs**

12
